# The novel function of the bZIP transcription factor VSF-1 in mediating fruit firmness in tomato

**DOI:** 10.1093/hr/uhag181

**Published:** 2026-04-30

**Authors:** Chunfeng Wang, Kangchi Zhang, Shouzheng Lv, Yifan Lin, Guanqing Su, Zimeng Liu, Xiahui Lin, Xiu Shu, Zhiren He, Mondher Bouzayen, Guojian Hu, Yanna Shi, Kunsong Chen

**Affiliations:** College of Agriculture and Biotechnology, Zhejiang University, Zijingang Campus, Hangzhou 310058, China; College of Agriculture and Biotechnology, Zhejiang University, Zijingang Campus, Hangzhou 310058, China; College of Agriculture and Biotechnology, Zhejiang University, Zijingang Campus, Hangzhou 310058, China; College of Agriculture and Biotechnology, Zhejiang University, Zijingang Campus, Hangzhou 310058, China; College of Agriculture and Biotechnology, Zhejiang University, Zijingang Campus, Hangzhou 310058, China; College of Agriculture and Biotechnology, Zhejiang University, Zijingang Campus, Hangzhou 310058, China; College of Agriculture and Biotechnology, Zhejiang University, Zijingang Campus, Hangzhou 310058, China; College of Agriculture and Biotechnology, Zhejiang University, Zijingang Campus, Hangzhou 310058, China; College of Agriculture and Biotechnology, Zhejiang University, Zijingang Campus, Hangzhou 310058, China; Laboratoire de Recherche en Sciences Végétales–Génomique et Biotechnologie des Fruits-UMR5546, Université de Toulouse, CNRS, Université Paul Sabatier, Institut Polytechnique de Toulouse, Auzeville Tolosan 31326, France; College of Agriculture and Biotechnology, Zhejiang University, Zijingang Campus, Hangzhou 310058, China; Zhejiang Provincial Key Laboratory of Horticultural Crop Quality Improvement, Zhejiang University, Zijingang Campus, Hangzhou 310058, China; The State Agriculture Ministry Laboratory of Horticultural Plant Growth, Development and Quality Improvement, Zhejiang University, Zijingang Campus, Hangzhou 310058, China; College of Agriculture and Biotechnology, Zhejiang University, Zijingang Campus, Hangzhou 310058, China; Zhejiang Provincial Key Laboratory of Horticultural Crop Quality Improvement, Zhejiang University, Zijingang Campus, Hangzhou 310058, China; The State Agriculture Ministry Laboratory of Horticultural Plant Growth, Development and Quality Improvement, Zhejiang University, Zijingang Campus, Hangzhou 310058, China; College of Agriculture and Biotechnology, Zhejiang University, Zijingang Campus, Hangzhou 310058, China; Zhejiang Provincial Key Laboratory of Horticultural Crop Quality Improvement, Zhejiang University, Zijingang Campus, Hangzhou 310058, China; The State Agriculture Ministry Laboratory of Horticultural Plant Growth, Development and Quality Improvement, Zhejiang University, Zijingang Campus, Hangzhou 310058, China

## Abstract

Fruit firmness is a critical quality trait primarily governed by the structure and composition of the cell wall. While extensive research has focused on firmness dynamics during fruit ripening, the regulatory mechanisms governing its establishment during early fruit development remain poorly understood. Previous studies have demonstrated that *VASCULAR SPECIFICITY FACTOR 1* (*VSF-1*), a member of the bZIP family, is activated *in vitro* by the key fruit softening regulator LOB1, suggesting its potential role in the regulation of fruit firmness. In this study, we generated both CRISPR-Cas9-mediated knockout and overexpression lines in tomato; functional analysis revealed that the loss of *VSF-1* function reduced fruit firmness, whereas its overexpression enhanced it, thereby establishing VSF-1 as a positive regulator of firmness. By integrating transcriptomic and DNA affinity purification sequencing profiling, we delineated the *VSF-1*-controlled gene regulatory network associated with cell wall remodeling and identified the β-galactosidase gene, *TBG6,* as a direct target*.* Our findings uncover a novel role for VSF-1 in the transcriptional regulation of tomato fruit texture, underscore the functional diversification within the *Solanaceae* bZIP subfamily I, and provide new insights into the genetic improvement of fruit quality.

## Introduction

Tomato (*Solanum lycopersicum*) represents not only a globally significant crop but also a well-established model system for studying climacteric fruit ripening. Its diploid genetics, relatively compact genome, efficient genetic transformation protocol, and short life cycle together make it an ideal organism for investigating fruit-related traits [1, 2]. Fruit firmness, one of the most critical quality attributes influencing consumer acceptance and shelf life, has been extensively studied in tomato, yielding significant insights into the genetic and molecular mechanisms underlying cell wall structure and texture regulation [3, 4].

Fruit firmness in tomato is a complex trait governed by multiple factors, including cell wall architecture, cell turgor pressure, and cuticular properties [5–7]. The plant cell wall is typically organized into the primary cell wall, the secondary cell wall, and the middle lamella between cells. The primary cell wall, which is most relevant to fruit softening, is primarily composed of cellulose, hemicellulose (predominantly xyloglucan in dicots), pectin, and various cell wall proteins [4, 7]. The primary load-bearing structure of the cell wall consists of cellulose microfibrils, which provide tensile strength, and xyloglucans that cross-link them at critical sites to form a coordinated framework [8]. Pectin, comprising homogalacturonan (HG), rhamnogalacturonan I (RGI), and rhamnogalacturonan II (RGII), occupies the interstices between cellulose layers [9]. Notably, in the middle lamella, pectin can form a gel-like matrix through Ca^2+^-mediated ‘egg-box’ structures, which is crucial for maintaining intercellular adhesion and overall tissue integrity [10]. Plant cell wall proteins are functionally categorized into enzymatic and nonenzymatic classes. Enzymatic proteins remodel the cell wall by catalyzing the hydrolysis or modification of wall polysaccharides. For example, pectate lyase (PL) and pectin methylesterase (PME) degrade and modify pectin polysaccharides [11, 12]. Nonenzymatic proteins serve distinct roles. For instance, expansins (EXPs) facilitate wall loosening [13], while other proteins, such as extensins (EXTs), glycine-rich proteins (GRPs), and arabinogalactan proteins (AGPs), provide mechanical strength through cross-linking and scaffold formation [14].

While the fundamental composition of the primary cell wall is well established, current research has predominantly focused on correlating fruit firmness with the abundance and architecture of its core components such as cellulose, hemicellulose, pectin, and structural proteins [12, 15, 16]. These studies have been instrumental in identifying key cell wall-modifying enzymes and cell wall proteins, such as polygalacturonases (PGs) [17], PLs [12], PMEs [11], β-galactosidases (β-Gals) [18], endoglucanases (EGs) [19, 20], xyloglucan endotransglucosylase/ hydrolases (XTHs) [21], and EXPs [13, 22]. The coordinated activities of these enzymes during fruit ripening lead to the controlled disassembly of the cell wall network and subsequent softening [15, 23]. In addition to the primary cell wall, the fruit cuticle also plays an important role in maintaining fruit integrity; it serves as a hydrophobic barrier composed of cutin, waxes, and polysaccharides [24]. It safeguards tissue integrity by limiting nonstomatal water loss, defending against pathogen invasion, and preventing physiological disorders such as fruit cracking [25]. During fruit development and ripening, cuticle deposition and remodeling occur concurrently with cell wall modifications; these processes collectively determine fruit quality and postharvest performance [26].

However, the complete hierarchical regulatory mechanism that governs the coordinated turnover of the cell wall and links its structure directly to fruit firmness remains to be fully elucidated. The spatiotemporal expression of these cell wall-modifying enzymes and proteins is precisely orchestrated by upstream transcription factors (TFs), which form a complex hierarchical regulatory network. Among these, MADS-box TFs such as RIN and NAC family members including NOR and NOR-LIKE1 have been identified as master regulators. They directly activate the promoters of key cell wall genes (e.g. *SlPG2a*, *SlPL*, *SlEXP1*), thereby coordinating the softening program alongside other ripening processes [27–29]. This regulatory framework is further elaborated by other key TFs, including MADS-box factors SlMBP3 and AP2/ERF factors (e.g. SlERF. G3-Like, SlERF. H5/H7), which regulate cell wall metabolism by targeting pectin, hemicellulose, and cellulose remodeling genes, collectively influencing fruit firmness at different stages [16, 30, 31]. In contrast, certain TFs exert more specific effects on fruit texture. For instance, SlLOB1 predominantly targets cell wall-related genes (e.g. *SlEXP1*, *SlCEL2*, *and SlPL*), governing the softening program with minimal impact on other ripening attributes [32]. In addition, SlBES1 has been identified as a promoter of fruit softening through its direct repression of PME gene (*PMEU1)*. *SlBES1* knockout (KO) mutants exhibited firmer fruit and extended shelf life during postharvest storage without compromising other quality traits [33].

Our previous work demonstrated that SlLOB1 strongly promotes the expression of the basic leucine zipper (bZIP) TF gene *VASCULAR SPECIFICITY FACTOR 1* (*VSF-1*) during fruit ripening, suggesting the involvement of *VSF-1* in fruit firmness regulation. Furthermore, VSF-1 regulates vascular-specific gene expression by binding to the promoter of *GRP1.8*, a gene encoding a glycine-rich structural protein in the cell wall [34, 35]. VSF-1 has also been shown to shuttle between the nucleus and the cytoplasm in response to hypo-osmotic stress [36], a finding conserved in its *Arabidopsis thaliana* homolog, VIP1 and AtbZIP29 [37], while the tobacco homolog, NtRSG, exhibits similar shuttling in response to gibberellin signaling [38]. Moreover, within the same bZIP subfamily I as *VSF-1*, certain bZIP members in *Arabidopsis*, such as *AtbZIP29*, have been implicated in cell wall organization in proliferating cells through the targeting of the xyloglucan endotransglucosylase gene *XTH9* and expansin-like genes [39]. Similarly, overexpression of *AtbZIP30* also alters the expression of genes involved in cell wall metabolism, cell cycle, and meristem activity [40]. Collectively, these functional links and conserved domain characteristics suggest a potential, yet uncharacterized, role for *VSF-1* in regulating analogous developmental and structural processes during fruit development in tomato.

In this study, we aimed to bridge this knowledge gap by functionally characterizing *VSF-1*, a member of the bZIP subfamily I in tomato. To elucidate its biological role, we generated both CRISPR-Cas9-mediated *VSF-1* KO mutants and *VSF-1* overexpressing lines. Phenotypic analysis revealed that the loss of *VSF-1* function resulted in a reduction in fruit firmness, unequivocally identifying *VSF-1* as a positive regulator of fruit firmness. Through comprehensive phenotyping and transcriptomic analyses of the *VSF-1* mutant, combined with genome-wide DNA affinity profiling (DAP-seq) of the VSF-1, we delineated the gene regulatory network governed by VSF-1. Our work identifies *VSF-1* as a new player in the transcriptional regulation of tomato fruit firmness, unveiling a previously unknown function of the bZIP subfamily I and offering novel insights into the genetic improvement of fruit quality.

## Results

### Expression profile and functional characterization of *VSF-1* in regulating fruit firmness in tomato

**Figure 1:**
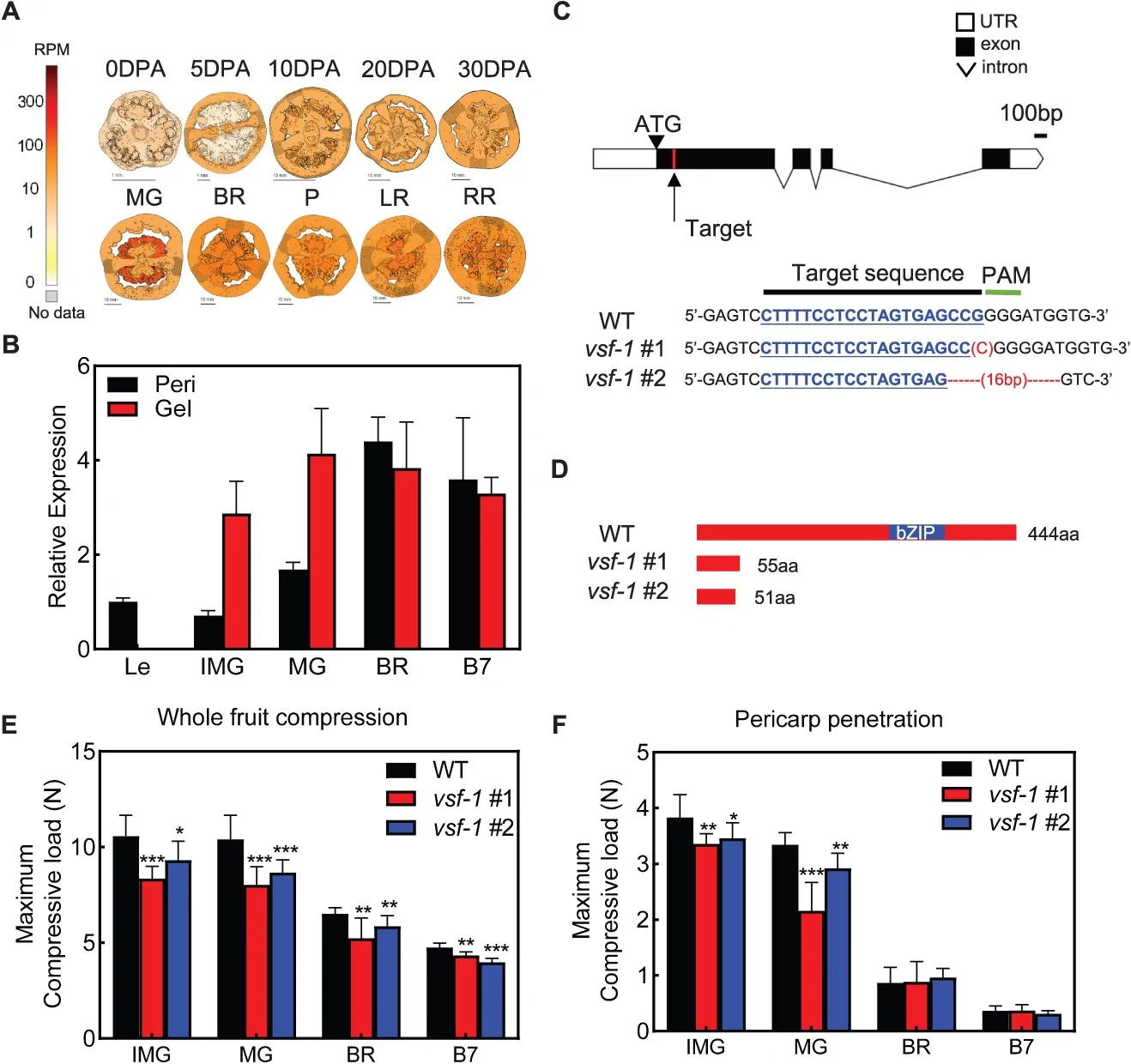
Knocking out of *VSF-1* leads to reduced fruit firmness in tomato. (A) Expression profile of *VSF-1* from the Tomato Expression Atlas (TEA) *S. lycopersicum* M82 RNA-seq database [41, 42] (https://tea.solgenomics.net/). P, pink; LR, light red; RR, red ripe; RPM, reads per million mapped reads. (B) Relative transcript level of *VSF-1* in leaf (Le), and fruit at different development stages, including immature green (IMG), mature green (MG), breaker (BR), breaker+7 days (B7) fruit in AC tomato cultivar, quantified by qRT-PCR. Data are presented as means ± SD (*n* ≥ 3). (C) Target site of *VSF-1* for CRISPR-Cas9 gene editing. Two independent knockout lines (*vsf-1* #1 and *vsf-1* #2) were generated. Target locus is indicated by a line in the exon of *VSF-1*. (D) Predicted VSF-1 protein structure in *vsf-1* mutants. The insertion/deletion mutations in *vsf-1* #1 and #2 result in predicted truncated proteins lacking the conserved bZIP domain (blue box). aa, amino acids. (E) Fruit firmness of WT and *vsf-1* mutant fruit measured by whole fruit compression at IMG, MG, BR, and B7 stages. Data are presented as means ± SD (*n* = 9). (F) Fruit firmness of WT and *vsf-1* mutant fruit measured by pericarp penetration at IMG, MG, BR, and B7 stages. Data are presented as means ± SD (*n* = 9). ^*^P < 0.05, ^**^P < 0.01, ^***^P < 0.001.

To investigate the expression profile of *VSF-1* during fruit development and ripening in tomato, we interrogated tissue-specific expression datasets from the Tomato Expression Atlas (TEA) database [41, 42] (https://tea.solgenomics.net/)**.** These data indicate that *VSF-1* is expressed in both pericarp and locular gel tissues throughout stages of fruit development and ripening, with higher transcript levels observed in the locular gel of mature green (MG) fruit (Fig. 1A). This expression pattern was consistent with the results obtained via RT-qPCR analysis in the ‘Ailsa Craig’ (AC) cultivar (Fig. 1B), suggesting a potential role for *VSF-1* in tomato fruit development.

To investigate the functional significance of *VSF-1* in tomato, CRISPR-Cas9-mediated genome editing was employed to disrupt its function. Two independent homozygous mutant lines, designated as *vsf-1* #1 and #2, were obtained. Sequencing analysis confirmed the presence of indels at the target sites of *VSF-1* in both KO lines. These mutations resulted in frameshifts and the generation of the premature termination codons, classifying both lines as KO mutants (Fig. 1C and D).

The *vsf-1* mutants (*vsf-1* #1 and *vsf-1* #2) exhibited no apparent growth defects compared to wild-type (WT) plants (Fig. S1A and B). Furthermore, the fruit appearance and major fruit ripening parameters—including the days to breaker stage, ethylene production, and soluble solids content—were unaffected (Fig. S1C–G). Similarly, postharvest assessment showed no significant difference in water loss between *vsf-1* mutants and WT fruit (Fig. S1H and I).

However, upon assessing fruit firmness, which was measured by whole fruit compression and pericarp penetration using a texture analyzer, the data revealed that *vsf-1* mutants exhibited significantly reduced whole fruit compressive firmness across all stages examined, from preripening through red ripe stages. In contrast, pericarp penetrative firmness was compromised specifically at the IMG and MG phases, but not at ripening stages (Fig. 1E and F). The whole-fruit compression assay reflects overall fruit firmness, whereas the pericarp penetration test reflects local tissue strength. The discrepancy between the two methods suggests that VSF-1 may regulate fruit firmness through multiple mechanisms, including both macroscopic structural integrity and cell wall properties. The most pronounced effects occurred at the MG stage, with fruit firmness decreasing by 17%–23% as measured by whole fruit compression and by 13%–35% as measured by pericarp penetration compared to WT (Fig. 1E and F). These data strongly indicate that *VSF-1* is mainly involved in fruit firmness regulation during the fruit development stage but weakly during fruit ripening stage.

**Figure 2:**
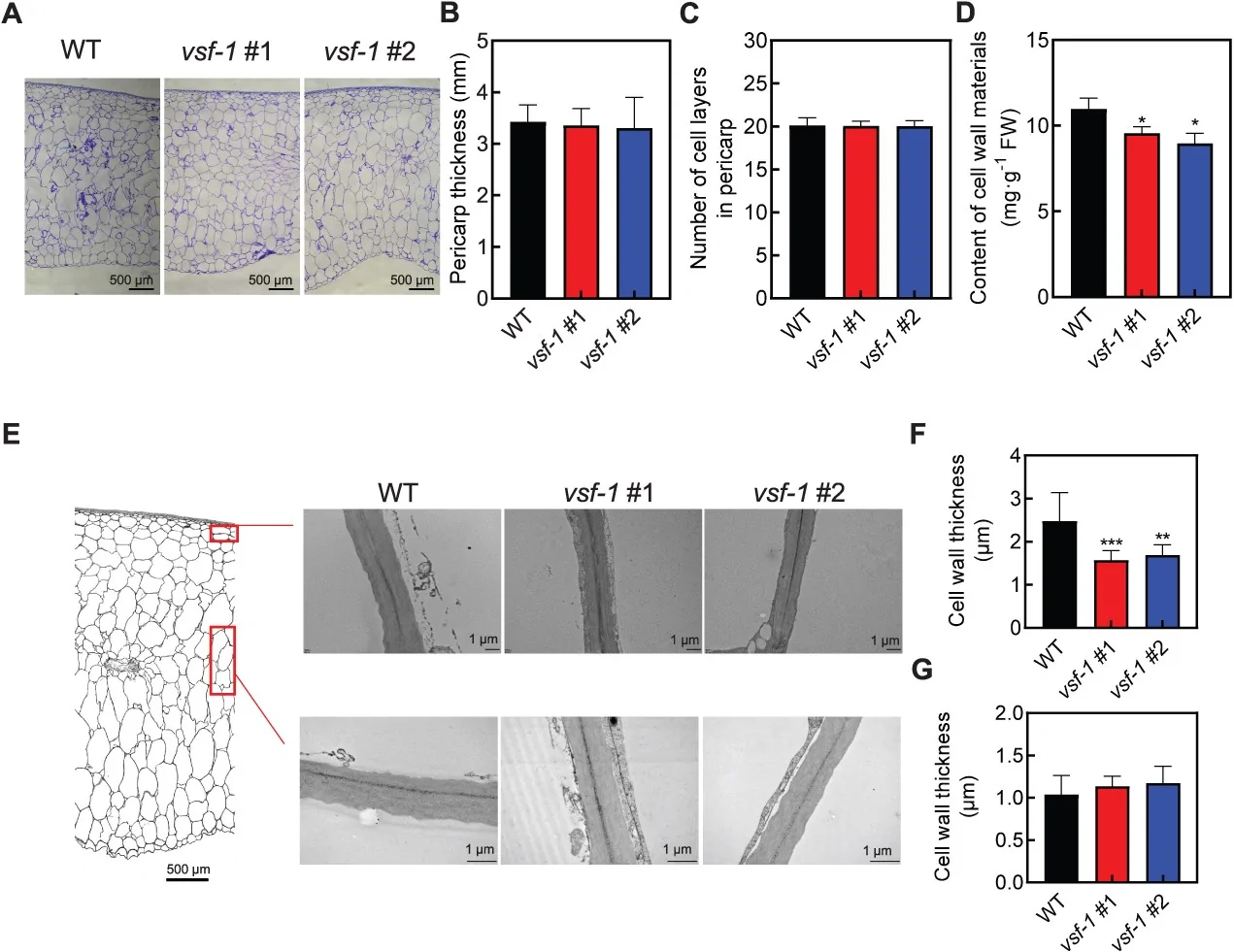
Altered cell wall ultrastructure and reduced cell wall thickness in the pericarp of *vsf-1* mutant fruits. (A) Microscopic analysis of fruit pericarp thickness in WT, *vsf-1* #1 and *vsf-1* #2 fruits. The scale bar represents 500 μm. (B) Quantification of pericarp thickness in WT and *vsf-1* mutants. Data are presented as means ± SD (*n* = 6). (C) Quantification of the number of cell layers counted across the pericarp for WT and *vsf-1* mutants. Data are presented as means ± SD (*n* = 6). (D) Content of cell wall material of fruit pericarp in WT and *vsf-1* mutant fruits. Data are presented as means ± SD (*n* = 3). (E) TEM images of the subepidermal (upper row) and central (lower row) regions of the pericarp. The scale bar represents 1 μm. (F-G) Quantitative analysis of cell wall thickness in the subepidermal (F) and central (G) cell regions shown in (E). Data are presented as means ± SD (*n* = 6). ^*^P < 0.05, ^**^P < 0.01, ^***^P < 0.001.

Fruit firmness is regulated at the tissue, cellular, and cell wall structure levels. To determine the underlying cause of the reduced firmness, light microscopy analysis of equatorial sections of MG fruit was performed, revealing no differences in pericarp thickness or the number of cell layers between *vsf-1* mutants and WT (Fig. 2A–C). These findings imply that the decreased fruit firmness in *vsf-1* mutants is not due to altered cell division or cell number. However, quantification of the cell wall materials (CWMs) in the fruit pericarp showed a significant reduction (13%–18%) in the KO mutants compared to the WT (Fig. 2D), suggesting specific alterations in the composition or architecture of the cell wall polymers. This was further supported by the transmission electron microscopy (TEM) analysis of the parenchyma cells. We observed a significant decrease (32%–37%) in cell wall thickness specifically in the subepidermal region of the KO pericarp, while no significant difference was observed in the central region (Fig. 2E–G). These data demonstrate that *VSF-1* regulates the cell wall structure of a specific cell layer of the pericarp, which may contribute to maintaining fruit firmness during the fruit development process in tomato.

### *VSF-1* overexpression enhances fruit firmness

**Figure 3:**
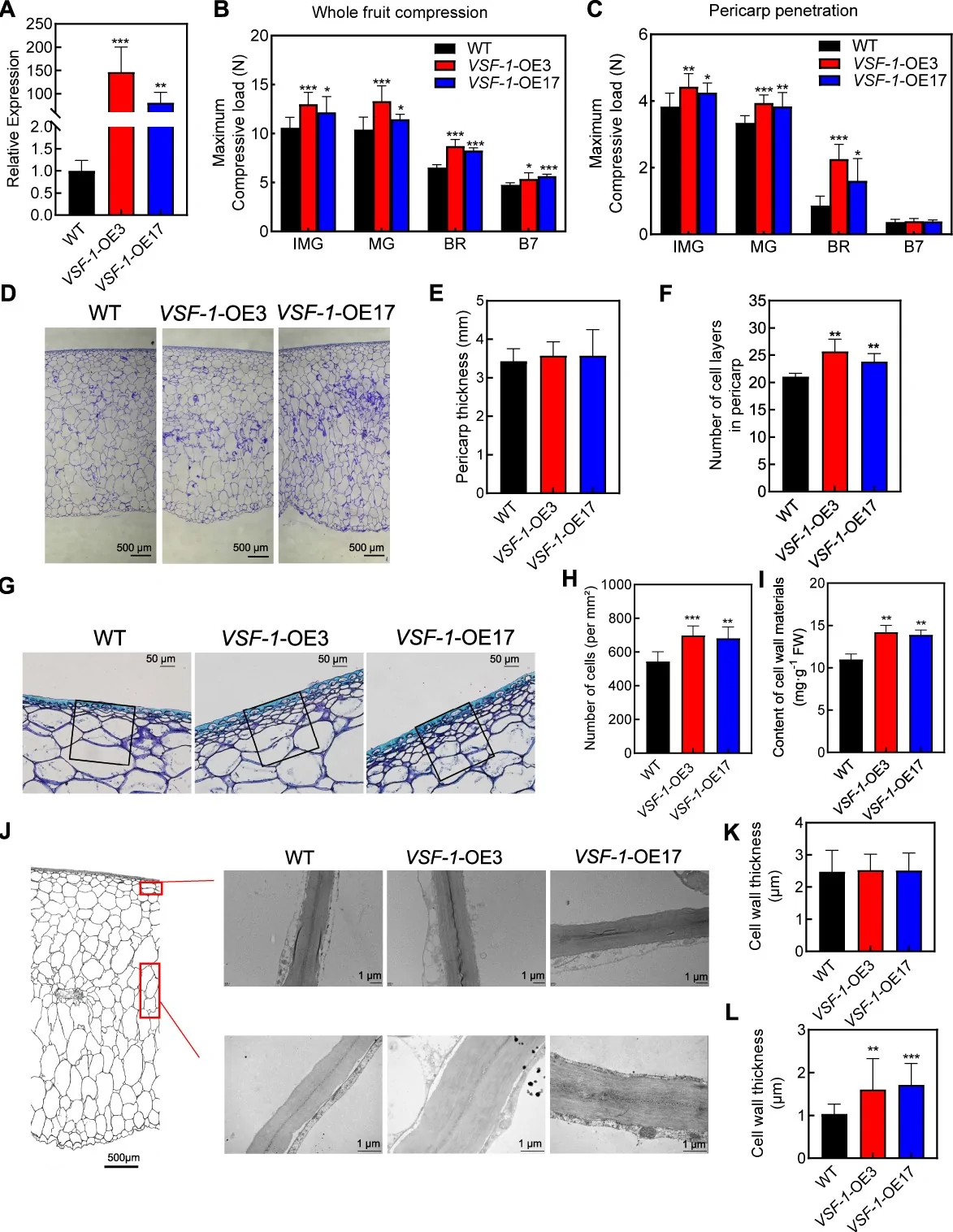
Increased fruit firmness and altered pericarp ultrastructure in *VSF-1*-overexpressing (*VSF-1*-OE) lines. (A) Relative expression level of *VSF-1* in young leaves of four-week-old WT and two *VSF-1*-OE lines, quantified by qRT-PCR. Data are presented as means ± SD (*n* ≥ 3). (B) Fruit firmness of WT and *VSF-1-*OE lines measured by whole fruit compression at IMG, MG, BR, and B7 stages. Data are presented as means ± SD (*n* = 9). (C) Fruit firmness of WT and *VSF-1-*OE lines measured by pericarp penetration at IMG, MG, BR, and B7 stages. Data are presented as means ± SD (*n* = 9). (D) Microscopic analysis of pericarp tissue section from WT, *VSF-1-*OE3, and *VSF-1-*OE17 fruits. The scale bar represents 500 μm. (E) Pericarp thickness in WT and *VSF-1*-OE lines. Data are presented as means ± SD (*n* = 6). (F) Quantification of the number of cell layers counted across the pericarp thickness for WT and *VSF-1*-OE lines. Data are presented as means ± SD (*n* = 6). (G) Microscopic images of subepidermal pericarp region in WT and *VSF-1*-OE tomato fruit. Black boxes indicate the defined areas used for cell counting analysis. Counting was performed for cells with >50% of their area falling within the box. The scale bar represents 50 μm. (H) Corresponding statistical analysis of cell numbers per unit area. Data are presented as means ± SD (*n* = 6). (I) Content of cell wall material of fruit pericarp in WT and *VSF-1*-OE lines. Data are presented as means ± SD (*n* = 3). (J) TEM images of the subepidermal (upper row) and central (lower row) regions of the pericarp. The scale bar represents 1 μm. (K-L) Quantitative analysis of cell wall thickness in the subepidermal (K) and central (L) cell regions shown in (J). Data are presented as means ± SD (*n* = 6). ^*^P < 0.05, ^**^P < 0.01, ^***^P < 0.001.

To further elucidate the function of *VSF-1*, we generated two independent overexpression lines in the tomato cultivar AC, designated as *VSF-1*-OE3 and *VSF-1*-OE17. These lines showed a substantial increase in *VSF-1* transcript levels, with *VSF-1*-OE3 exhibiting an increase to 146-fold and OE17 showing an increase to 81-fold compared to WT plants (Fig. 3A). Both overexpression lines exhibited a reduction in plant height. At four weeks after germination, the plant height was reduced by approximately 30%–33% compared to WT (Fig. S2A and B). However, fruit weight and size were not affected in the OE lines (Fig. S2C and D).

In contrast to the reduced firmness observed in *vsf-1* mutants, fruit firmness was significantly increased at all fruit developmental stages examined in the OE lines compared to WT plants, as measured by whole-fruit compression (Fig. 3B). In line with this, pericarp penetrative firmness was also elevated in the OE lines, including at the IMG, MG, and BR stages (Fig. 3C).

Light microscopy analysis of pericarp sections revealed that the enhanced firmness in *VSF-1*-overexpressing fruit was associated with an increased number of cells in the pericarp, without, however, a significant change in the overall pericarp thickness (Fig. 3D and E). The number of cell layers was increased by 13%~22% in the OE lines compared to WT (Fig. 3F). Further examination of the subepidermal region of pericarp tissue demonstrated that the increased number of cells in the OE lines is primarily localized to collenchyma tissue (Fig. 3G and H). In line with this feature, we also observed that the CWM content was elevated by 27%–30% in the pericarp of *VSF-1*-overexpressing lines compared to WT plants (Fig. 3I). TEM analysis revealed a significant increase in cell wall thickness in the central parenchyma region of the pericarp in the overexpression lines compared to WT (Fig. 3J and L), while no significant difference was observed in the subepidermal parenchyma region (Fig. 3J and K). Collectively, these data suggest that the enhanced fruit firmness in the overexpression lines arises from multiple factors, including a marked increase in the number of collenchyma cells and the thickened cell walls in the central parenchyma cells.

Unexpectedly, fruit from the overexpression lines harvested at the B3 stage and stored at room temperature for 10, 20, 30, and 40 days postharvest exhibited a 2-fold increase in water loss compared to WT at all time points (Fig. S2D and E). To investigate the underlying cause, Oil Red O staining was employed to examine cuticular properties. *VSF-1*-OE fruits exhibited significantly increased cuticular invagination compared to WT (Fig. S2F and G). Additionally, consistent with the accelerated water loss in OE lines, toluidine blue staining revealed intensive dye penetration on the fruit surface, a phenomenon not observed in WT or KO fruit (Fig. S2H). Based on these findings, we hypothesize that the overexpression of *VSF-1* enhances cuticle permeability, thereby facilitating excessive transpiration and explaining the increased rate of water loss observed in *VSF-1* overexpressing fruit.

### Transcriptional regulation of cell wall metabolism by the VSF-1 transcription activator

**Figure 4:**
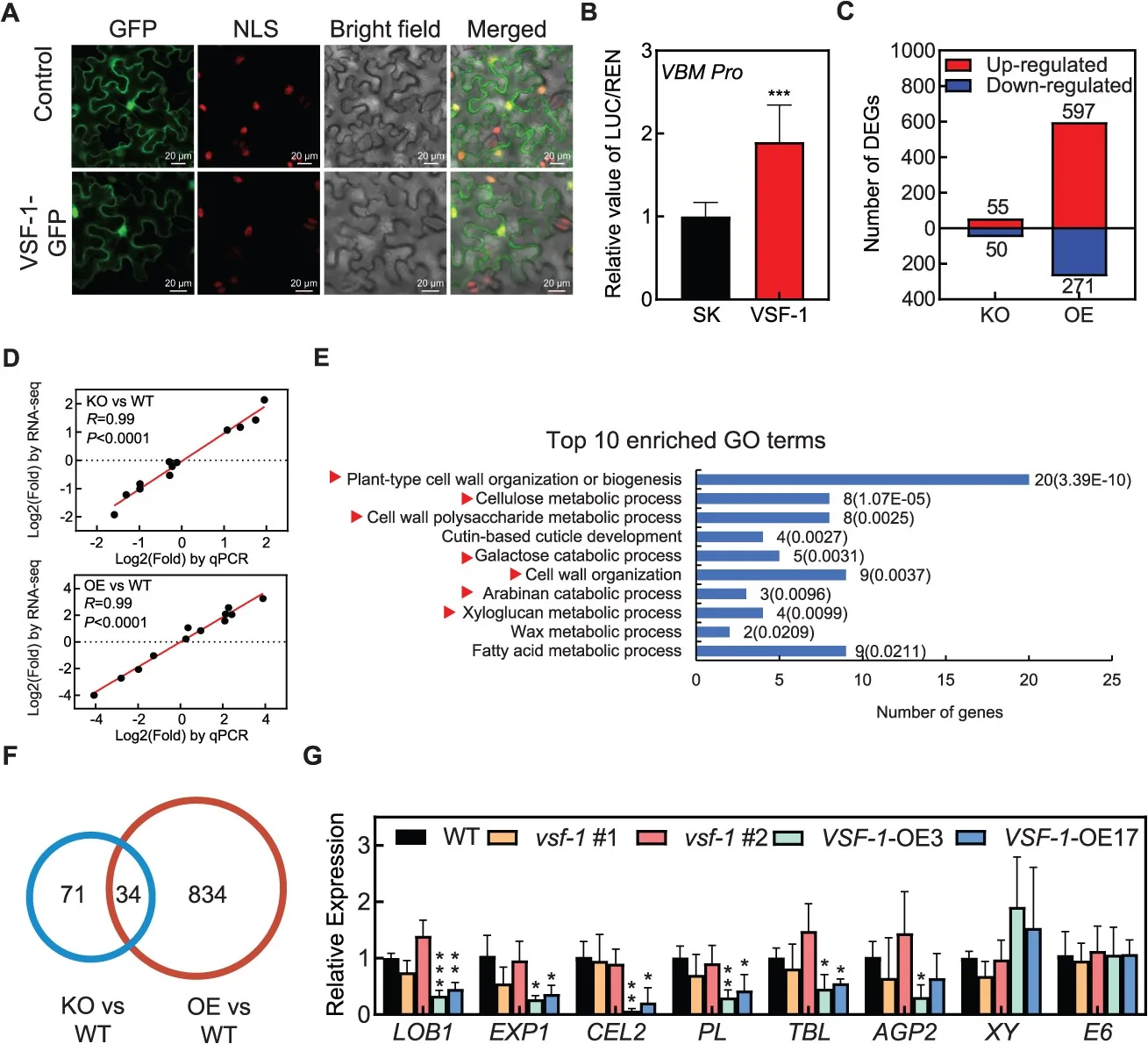
Transcriptomic and functional analyses reveal that *VSF-1* functions as a nuclear-localized transcriptional activator regulating cell wall-associated genes. (A) Subcellular localization of VSF-1. VSF-1 fused with GFP was transiently expressed in *N*. *benthamiana* leaf epidermal cells via agroinfiltration. GFP alone served as a negative control. Images were taken 2 days postagroinfiltration. (B) Transient transcriptional activity of VSF-1 using a dual-luciferase system. The activity of VSF-1 was assessed by its ability to activate a promoter containing the VSF-1 binding motif (VBM) fused to the firefly luciferase gene (*LUC*). The relative LUC activities normalized to the REN activity are shown (LUC/REN). SK (empty vector) serves as the negative control. Data are presented as means ± SD (*n* = 3). (C) Number of differentially expressed genes (DEGs) between *vsf-1* mutants and WT, and between *VSF-1*-OE lines and WT. (D) Correlation analysis of RNA-seq and qRT-PCR data. The statistical significance of the correlation between the expression fold change (Log2Fold) measured by RNA-seq and that measured by qRT-PCR was determined by a two-sided Pearson’s correlation test. Upper panel: *KO vs. WT*. Lower panel: *OE vs. WT*. The correlation coefficient (R) and *P-value* are indicated for both comparisons. (E) Top 10 enriched GO Biological Process terms from the list of DEGs in *VSF-1*-OE lines. Arrows indicate GO terms associated with cell wall organization or biogenesis. (F) Venn diagram of DEGs common to the *VSF-1* knockout (KO) and overexpression (OE) lines when compared to WT. (G) Relative expression levels of *LOB1* and its downstream target genes in WT and mutant fruits, quantified by qRT-PCR. Data are presented as means ± SD (*n* = 3). ^*^P < 0.05, ^**^P < 0.01, ^***^P < 0.001.

Subcellular localization analysis revealed that the GFP-tagged VSF-1 protein was predominantly localized in the nucleus (Fig. 4A) with a relatively weak signal also observed at the plasma membrane and in the cytoplasm, consistent with previous reports on bZIP subfamily I members [37]. To validate the transcriptional activity of VSF-1 in tomato, we employed a dual-luciferase (LUC) reporter assay. A synthetic promoter was designed, incorporating nine repeats of the predicted binding motif for AtbZIP30 (the closest *Arabidopsis* homolog to VSF-1), which was obtained from the JASPAR database [43] (JASPAR ID: MA1744.1). This motif was coupled with a minimal 35S promoter. Our results demonstrate that VSF-1 significantly activates the synthetic promoter, confirming its function as a transcriptional activator (Fig. 4B). Collectively, the predominant nuclear accumulation and robust transactivation capacity corroborate the role of VSF-1 as a TF.

To elucidate the molecular mechanism by which VSF-1 regulates fruit firmness, and considering the pericarp-level alterations in *vsf-1* mutants and overexpression lines, a global transcriptomic analysis was performed on the pericarp tissue of MG fruit from these lines and the WT. In the KO mutants, 105 genes were differentially expressed genes (DEGs, cutoff: false discovery rate [FDR] < 0.05; ratio > 1.5 or < 0.67), comprising 55 upregulated and 50 downregulated genes. By contrast, the overexpression lines exhibited more extensive transcriptomic changes, with 597 genes upregulated and 271 downregulated (Fig. 4C). To validate these datasets, qRT-PCR analysis was performed for 12 randomly selected DEGs, all of which displayed expression changes consistent with the RNA-seq data in both *vsf-1* mutant and *VSF-1*-OE, confirming the accuracy of the RNA-seq results for subsequent analysis (Fig. 4D).

Gene Ontology (GO) enrichment analysis of DEGs between *VSF-1*-overexpressing fruit and WT revealed significant enrichment of biological processes related to cell wall organization, including ‘plant-type cell wall organization or biogenesis’, ‘cellulose metabolic process’, and ‘cell wall polysaccharide metabolic process’ (Fig. 4E). The large number of DEGs involved in cell wall biosynthesis, remodeling, hydrolysis, and cell wall signaling indicates active and dynamic cell wall restructuring in the OE line (Table S1), consistent with its enhanced fruit firmness. Comparative transcriptomic analysis identified 34 DEGs common to the *vsf-1* KO and overexpression (OE) lines when compared to WT. (Fig. 4F). Among these genes, five cell wall–associated DEGs exhibited opposite expression trends between the KO and overexpression fruits (Table 1). These include *PMEU1,* which catalyzes the demethylesterification of pectin in fruit [44], two *XTH* family genes likely involved in cell wall rearrangement [21, 45], and a *CC1* gene whose *Arabidopsis* homologue can interact with cellulose synthase complex for cell wall remodeling [46]. Collectively, the transcriptomic data indicate that *VSF-1* mediates fruit firmness in tomato primarily through cell wall related genes. In addition, GO terms including ‘cutin-based cuticle development’ and ‘wax metabolic process’ were also significantly enriched (Fig. 4E), consistent with the observation of a thicker cuticle layer in *VSF-1*-overexpressing fruit (Fig. S2F and G).

**Table 1:** Cell wall-related DEGs in both *VSF-1* knockout and overexpression lines.

**Gene ID**	**Annotation**	**KO fold**	**OE fold**	**Blast hit in *Arabidopsis***	**Function**
Solyc03g123630.4	*PMEU1* [11, 33])	0.43	5.95	AT3G14310, PME3	Catalyzes the demethylesterification of pectin
Solyc01g099630.4	Xyloglucan endotransglucosylase/hydrolase 1 (*XTH1* [21]*)*	0.56	4.24	AT5G13870, XTH5	Regulates cell wall dynamic remodeling
Solyc04g008210.2	ETAG-A3 (*XTH8*)	0.50	3.14	AT2G01850, XTH27	Regulates cell wall dynamic remodeling
Solyc04g078750.3	Late embryogenesis abundant protein	0.50	2.98	AT1G45688, CC1	Interacts with the cellulose synthase complex and microtubules in *Arabidopsis*
Solyc03g115710.1	Receptor-like protein kinase THESEUS 1	0.32	2.68	AT5G61350, CAP1	Influence vesicle trafficking and the cytoskeletal arrangement involved in cell wall synthesis

Notably, we found that the transcript abundance of *LOB1*, an upstream regulator of *VSF-1*, was significantly suppressed in the overexpression lines, a result validated by our RT-qPCR analysis (Figs 4G and S3). This suggests a negative feedback loop where VSF-1 activity regulates LOB1 expression. Strikingly, out of seven downstream target genes of LOB1 involved in cell wall remodeling, four genes were significantly downregulated in OE lines (Figs 4G and S3). These included: *Expansin 1* (*EXP1)*， which is involved in cell wall loosening [15, 22]; *Cellulase 2* (*CEL2),* responsible for cell wall polysaccharide breakdown [15, 19]; *Pectin lyase (PL)，*which directly degrades pectin [12]; and *Trichome birefringence-like 36* (*TBL)*, potentially involved in pectin *O*-acetylation [47].

Given that *EXP1, CEL2*, and *PL* are major contributors to fruit firmness loss [12, 15, 48], these data strongly suggest that *VSF-1* maintains fruit firmness not only by activating cell wall genes, but also by repressing the transcriptional activity of LOB1 and, consequently, suppressing the expression of its downstream cell wall degradation genes.

### Global profiling of potential targets of VSF-1 using DAP-seq approach

**Figure 5:**
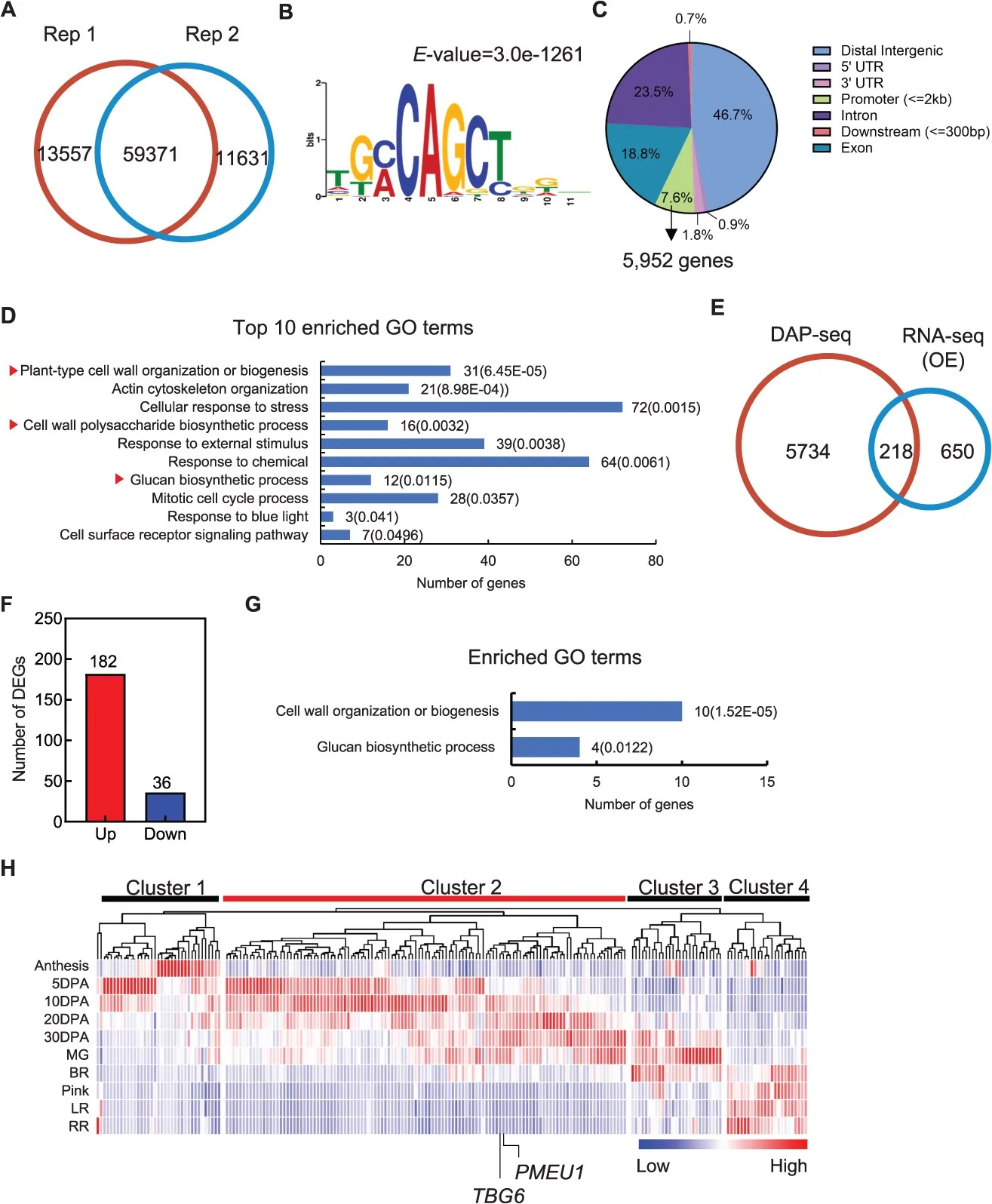
Genome-wide identification of VSF-1 binding sites through DAP-seq. (A) DAP-seq using two biological replicates reveals 59371 high confidence VSF-1 binding peaks. (B) Identification of enriched motif in VSF-1 binding sequences using MEME software [49]. (C) Distribution of VSF-1 binding peaks within tomato genic regions. (D) Top10 enriched GO Biological Process terms for the 5952 genes whose promoters contain VSF-1 binding sites. Arrows indicate GO terms associated with cell wall organization or biogenesis. (E) Venn diagram of overlapping genes between RNA-seq and DAP-seq data. (F) Up-regulation and down-regulation distribution of 218 overlapping target genes in (E). (G) Two enriched GO terms for the 218 predicted direct target genes regulated by VSF-1. (H) Cluster analysis of expression patterns of VSF-1 target genes during fruit development. Examples of known cell wall genes, such as *TBG6* and *PMEU1*, are highlighted to show the correlation between their expression and VSF-1 regulation.

To identify the genome-wide binding sites of VSF-1 and elucidate its regulatory network, DNA affinity purification sequencing (DAP-seq) was performed using MG fruit. To ensure robust and reproducible results, DAP-seq was conducted with two biological replicates, incorporating a standard input control library, followed by high-throughput sequencing. This analysis successfully identified 59 371 enriched peaks across the two biological replicates (Fig. 5A). A *de novo* motif prediction analysis using MEME tools [49] revealed the most significantly enriched motif to be ‘GCCAGCT’, which is highly similar to the known binding motif for the bZIP subfamily I factor bZIP29 [39], thereby validating the reliability of our DAP-seq data (Fig. 5B).

By intersecting DAP-seq peaks with gene promoter regions, a total of 5952 genes were identified (Fig. 5C). GO enrichment analysis of these genes revealed significant association with GO terms including ‘plant-type cell wall organization or biogenesis’, ‘cell wall polysaccharide biosynthetic process’, and ‘glucan biosynthetic process’ (Fig. 5D), consistent with the transcriptomic data analyzed in *VSF-1* mutant fruit. By integrating the transcriptomic data of *VSF-1* overexpression lines with DAP-seq results, we identified an overlap of 218 genes that are both DEG and directly bound by VSF-1 (Fig. 5E). Among these potential direct targets, 182 were upregulated and 36 were downregulated at the transcriptional level in the overexpression lines (Fig. 5F), a result consistent with VSF-1 functioning primarily as a transcriptional activator. Notably, GO analysis of these 218 genes again demonstrated that the preferential enrichment of plant cell wall organization processes is significantly enriched (Fig. 5G). These integrated data strongly support the conclusion that VSF-1 directly controls cell wall-related genes and processes in mediating fruit firmness in tomato.

Using tissue-specific expression data from the TEA database [41, 42], we performed cluster analysis on the expression patterns of the 218 potential direct target genes across various developmental stages of tomato fruit. These genes were grouped into four distinct clusters. Clusters 1 and 4 contained genes expressed specifically at the very early and ripening stages of fruit development, respectively. Clusters 2 and 3 genes were predominantly expressed during the fruit expansion phase, suggesting their specific involvement in cell wall remodeling at this critical stage (Fig. 5H). Among the cell wall-related genes identified in Clusters 2 and 3, two previously characterized genes, *TBG6* [18] and *PMEU1* [11, 33], were found to be potentially associated with fruit firmness (Fig. 5H, Table 2).

**Table 2:** Cell wall-related DEGs identified in clusters 2 and 3 from the cluster analysis.

**Gene ID**	**Annotation**	**OE fold**	**DAP fold**	**Blast hit in *Arabidopsis***	**Function**
***Cluster 2***					
Solyc08g083310.3	Endo-1,3-β-glucanase	2.14	6.29	AT5G42100, BG_PPAP	Involved in plasmodesmal callose degradation
Solyc04g007160.1	Alpha-xylosidase 1-like	0.49	8.21	AT1G68560, XYL1	Degrade XyG by trimming side chains
Solyc02g092790.3	Arabinogalactan	4.22	7.88	N/A	AGP
Solyc09g072820.4	Cellulose synthase	12.42	11.85	AT5G44030, CesA4	Involved in secondary cell wall biosynthesis
Solyc04g071650.4	Cellulose synthase	6.09	10.2	AT5G64740, CesA6	Related to primary cell wall biosynthesis
Solyc08g061100.3	Cellulose synthase	2.41	18.62	AT4G32410, CesA1	Related to primary cell wall biosynthesis
Solyc04g081300.4	Endoglucanase	2.75	9.21	AT1G19940, GH9B5	Cleave internal β-1,4-glycosidic bonds in cellulose and other β-glucans
Solyc11g005150.3	Extensin X55687	5.16	15.3	AT1G12040, LRX1	A chimeric leucine-rich repeat/extensin protein
Solyc07g053540.1	Fasciclin-like AGP 9	9.5	11.06	AT1G03870, FLA9	A subclass of AGPs
Solyc12g015690.3	Fasciclin-like AGP 1	5.69	8.68	AT5G55730, FLA1	A subclass of AGPs
Solyc02g065530.4	Hexosyltransferase	39.47	5.15	AT3G50760, GATL2	Involved in cell wall biosynthesis
Solyc05g009820.4	Hexosyltransferase	2.71	19.55	AT1G13250, GATL3	Participate in pectic polysaccharide synthesis
Solyc04g078750.3	Late embryogenesis abundant protein	2.98	11.81	AT1G45688, CC1	Interact with the cellulose synthase complex and microtubules
Solyc03g123620.4	Pectinesterase	10.45	10.26	AT1G53840, PME1	Catalyze the demethylation of pectin
Solyc03g123630.4	*PMEU1*	5.95	9.1	AT3G14310, PME3	Catalyze the demethylation of pectin
Solyc06g051960.3	Pectinesterase	4.07	9.71	AT3G49220, PME34	PME involved in transpiration, heat tolerance
Solyc07g042390.3	Plant invertase/PME inhibitor superfamily protein	2.86	8.05	AT1G62770, PMEI9	PME inhibitor
Solyc03g083770.1	Plant invertase/PME inhibitor superfamily protein, putative	0.19	5.21	AT5G62360, PMEI13	PME inhibitor
Solyc07g062130.3	Trifunctional UDP-glucose 4,6-dehydratase/UDP-4-keto-6-deoxy-D-glucose 3,5-epimerase/UDP-4-keto-L-rhamnose-reductase RHM1	3.05	12.33	AT1G78570, RHM1	A UDP-L-rhamnose synthase involved in the biosynthesis of rhamnose
Solyc01g111540.4	Beta-galactosidase	22.87	9.39	AT2G16730, BGAL13	Putative beta-galactosidase
Solyc02g084720.3	Beta-galactosidase 6 (*TBG6*)	4.52	14.28	AT4G36360, BGAL3	Putative beta-galactosidase
Solyc10g006870.1	Receptor-like protein kinase THESEUS 1	3.02	12.97	AT5G54380, THE1	Encode THESEUS1 (THE1), a receptor kinase required for cell elongation during vegetative growth
Solyc03g031800.3	Xyloglucan endotransglucosylase/hydrolase	9.81	9.73	AT1G11545, XTH8	Regulate cell wall dynamic remodeling
Solyc01g067930.4	Xyloglucan 6-xylosyltransferase 1	2.03	9.71	AT3G62720, XXT1	Add the xylosyl residue from UDP-xylose onto a glucan backbone chain
Solyc06g074670.4	UDP-apiose/UDP-xylose synthase	4.13	18.35	AT1G08200, AXS2	Synthesize sugar donors for cell wall polymers
***Cluster 3***					
Solyc08g007310.3	Exostosin-like	2.21	7.45	AT5G33290, XGD1	Involved in pectin biosynthesis
Solyc01g006360.4	Callose synthase 9	1.83	13.3	AT3G07160, GSL10	May be involved in the synthesis of the cell wall component callose
Solyc07g055990.3	Xyloglucan endotransglucosylase/hydrolase	13.07	7.92	AT3G23730, XTH16	Regulate cell wall dynamic remodeling

Additionally, of the four cuticle-associated DEGs identified in *VSF-1*-OE transcriptomic analysis, only *GPAT6,* a cutin biosynthesis gene [50]), was detected as a potential direct target of VSF-1 through our DAP-seq analysis (Fig. S4A and B). Dual-LUC assays confirmed that VSF-1 could activate the *GPAT6* promoter, albeit with a modest induction level (Fig. S4C). These data suggest that while *GPAT6* is a direct target, the expression changes observed in the majority of cuticle and wax-related genes likely occur through indirect regulatory mechanisms.

### VSF-1 modulates the expression of *TBG6* and *PMEU1* to regulate fruit firmness

**Figure 6:**
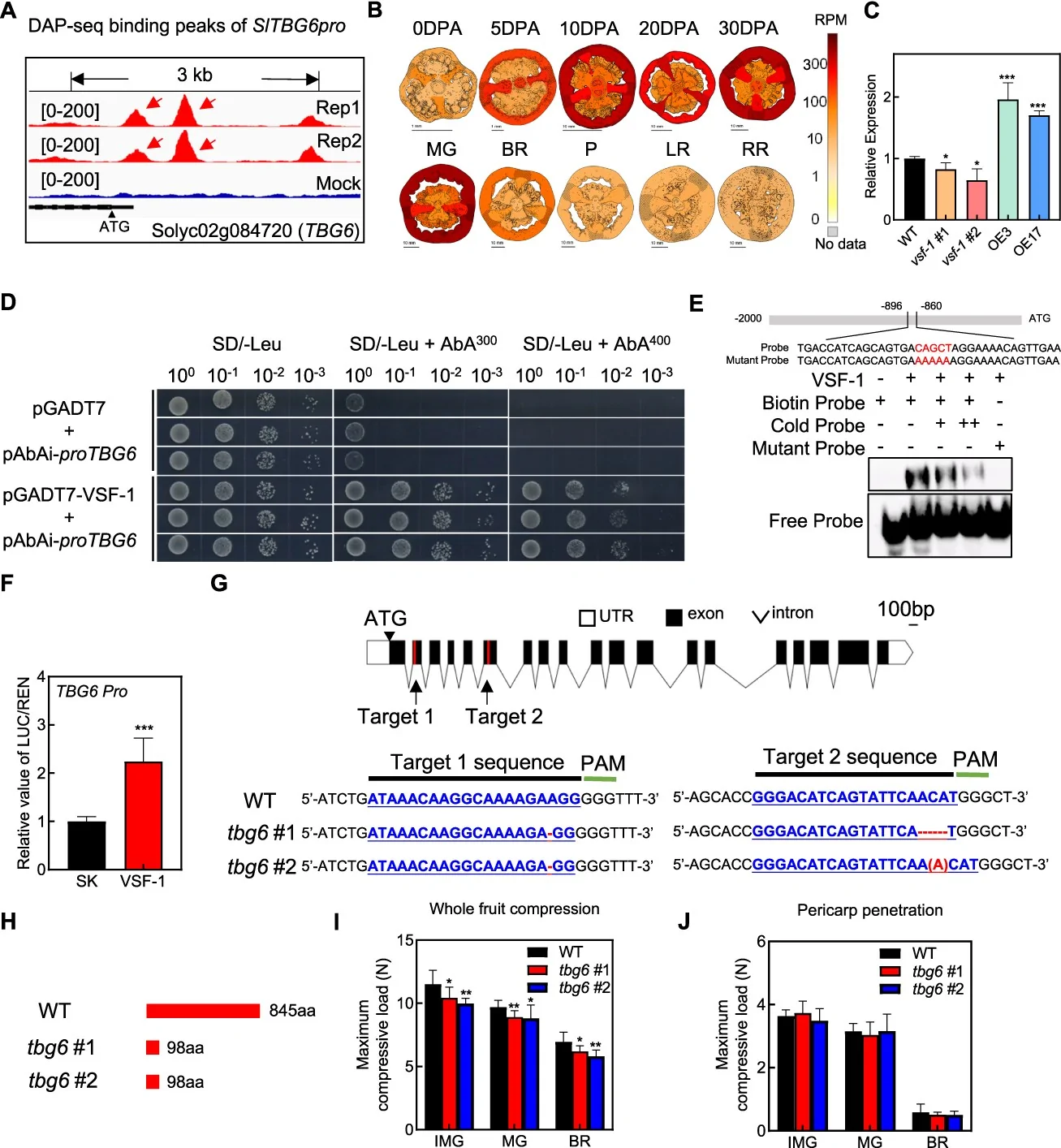
VSF-1 directly activates the transcription of *TBG6*, and knocking out *TBG6* reduces fruit firmness. (A) VSF-1 binding peaks present in the promoter region of *TBG6* as determined by DAP-seq. Arrows indicate the high-confidence binding peaks in the promoter region (<3 kb upstream of the ATG start codon). (B) Expression pattern of the *TBG6* gene during fruit development. Expression profile from TEA database [41, 42] (https://tea.solgenomics.net/). P, pink; LR, light red; RR, red ripe; RPM, reads per million mapped reads. (C) Relative transcript abundance of *TBG6* in the pericarp tissue of WT, *vsf-1* mutants and *VSF-1*-OE lines, quantified by qRT-PCR. Data are presented as means ± SD (*n* = 3). (D) Yeast one hybrid assay reveals VSF-1 binding to the *TBG6* promoter. pGADT7-VSF-1 served as the prey and pAbAi-*proTBG6* was used as the baits. Empty pGADT7 served as the negative control. The transformants were selected on SD/−Leu medium with AbA. (E) EMSA results show that the direct binding of VSF-1 to the *TBG6* promoter. Symbols (+), (−), and (++) indicate the presence, absence, or increasing concentrations of the respective components. The full gel image is provided in Fig. S5. (F) Transient transcriptional activity assay in tobacco leaves indicated the activation of the *TBG6* promoter by VSF-1. The relative LUC activities normalized to the REN activity are shown (LUC/REN). SK (empty vector) serves as the negative control. Data are presented as means ± SD (*n* = 4). (G) Targeting strategy for *TBG6* using CRISPR-Cas9 gene editing and analysis of homozygous mutant alleles. Target sites are indicated by lines in the exons of *TBG6*. (H) Predicated TBG6 protein structure in *tbg6* knockout lines. The mutations in *tbg6* #1 and #2 result in predicted truncated proteins lacking the conserved domain. The truncation is expected to cause a loss-of-function. (I) Fruit firmness of WT and *tbg6* mutant fruit measured by whole fruit compression at IMG, MG, and BR stages. Data are presented as means ± SD (*n* = 9). (J) Fruit firmness of WT and *tbg6* mutant fruit measured by pericarp penetration at IMG, MG, and BR stages. Data are presented as means ± SD (*n* = 9). ^*^P < 0.05, ^**^P < 0.01, ^***^  *P* < 0.001.

To investigate the molecular mechanisms downstream of VSF-1, *TBG6* was prioritized for in-depth characterization as it represented the most compelling candidate overlapping our DAP-seq and RNA-seq datasets with an established link to fruit texture in the early fruit developmental stage. *TBG6* encodes a β-galactosidase and was previously been demonstrated to be associated with reduced fruit firmness at early developmental stages in antisense-suppressed tomato fruit [18]. Our DAP-seq analysis revealed a significant enrichment of VSF-1 binding peaks within the promoter region of *TBG6* (Fig. 6A). Furthermore, expression data extracted from the TEA database [41, 42] indicated that *TBG6* is predominantly expressed during preripening fruit developmental stages, suggesting an essential function in early fruit growth (Fig. 6B). Subsequent qRT-PCR analysis demonstrated that the transcript level of *TBG6* was significantly downregulated in *vsf-1* mutants and upregulated in overexpression lines (Fig. 6C), validating the *VSF-1-TBG6* transcription regulatory module in tomato fruit. To confirm this direct interaction, yeast one hybrid (Y1H) analysis showed that VSF-1 binds to the *TBG6* promoter (Fig. 6D). Consistent with this, electrophoretic mobility shift assay (EMSA) using a probe derived from a DAP-seq peak with a high fold-enrichment confirmed the direct binding of VSF-1 to this sequence (Fig. 6E). Additionally, dual-LUC assays confirmed that VSF-1 exerts a transactivation effect on the promoter of *TBG6* (Fig. 6F).

To further elucidate the contribution of *TBG6* to the control of fruit firmness, we employed CRISPR-Cas9-mediated genome editing in the tomato cultivar AC. Two edited lines were generated, both harboring premature stop codons leading to a frameshift in the TBG6 protein (Fig. 6G and H); consequently, these two lines were designated as KO lines. Consistent with the reduced firmness observed in *VSF-1* KO lines, the *tbg6* lines also displayed a moderate reduction in firmness at the IMG, MG, and BR stages when measured by whole fruit compression (Fig. 6I). However, pericarp penetration firmness showed no change (Fig. 6J), suggesting the involvement of additional regulatory factors. Collectively, these data demonstrate that VSF-1 likely targets *TBG6* directly to positively modulate fruit firmness in tomato.

DAP-seq analysis further revealed a significant binding peak of VSF-1 within the promoter region of *PMEU1* (Fig. S6A)*. PMEU1* encodes a PME and has been previously implicated in the regulation of fruit firmness [11, 44]. Regarding its established role in cell wall modification, *PMEU1* expression is elevated in developing tomato fruit, and subsequently declines during the ripening stages (Fig. S6B), suggesting that *PMEU1* likely contributes to the maintenance of cell wall integrity during fruit expansion. Furthermore, RT-qPCR analysis demonstrated that *PMEU1* transcript levels were significantly downregulated in the *vsf-1* mutant and upregulated in the overexpression line (Fig. S6C). EMSA confirmed that VSF-1 binds directly to the promoter of *PMEU1 in vitro* (Fig. S6D). However, transient transactivation assays using a dual-LUC reporter system failed to exhibit significant induction of the *PMEU1* promoter by VSF-1 (Fig. S6E). These data suggest that the specific cellular context or essential co-regulators required for the VSF-1-mediated transactivation of *PMEU1* might be absent in the heterologous transient expression system. Given that members of the bZIP family frequently functions as homo- or heterodimers [51], the absence of appropriate co-regulators in the tobacco transient system is a plausible explanation for this observation. To identify potential partners, we screened for bZIP-family genes that were both highly expressed in our transcriptomic datasets (Fragments Per Kilobase of exon model per Million mapped fragments [FPKM >10]) and strongly co-expressed with *VSF-1* across tomato tissues (Pearson correlation coefficient > 0.6, based on the TEA database [41, 42]); these candidates are listed in Table S2 as putative VSF-1-interacting bZIPs.

## Discussion

### Novel function of VSF-1 in mediating fruit firmness during the developmental stage

Firmness contributes to mechanical strength and tissue integrity, thereby enhancing resistance to environmental mechanical stresses and pathogen infection [12, 52]. Firmness is generally maintained at a high level during fruit expansion but undergoes a precipitous decline at the onset of ripening [4, 53]. In this study, through the functional characterization of VSF-1, a member of the bZIP subfamily I, we elucidated a novel role for this TF as a positive regulator of fruit firmness in tomato. Consistent with the high expression of *VSF-1* in early developing fruit, phenotypic analysis demonstrated that *vsf-1* mutants exhibited reduced firmness from the IMG to MG stages, whereas its overexpression resulted in enhanced firmness. The observed modulation of firmness was directly correlated with changes in cell wall architecture, specifically through increased CWM content in overexpression lines, and a corresponding reduction in *vsf-1* mutants.

Mechanistically, integrated transcriptomic and genome-wide DAP-seq analyses conclusively revealed that VSF-1 exerts its influence by primarily targeting cell wall-associated genes. The VSF-1 regulatory network is significantly enriched in processes such as ‘cell wall organization’ and ‘cell wall polysaccharide metabolic process’. Notably, several key cell wall genes—including those encoding β-galactosidase (*TBG6), PMEU1*, and xyloglucan endotransglucosylase/hydrolases (*XTH1* and *XTH8*)—were significantly suppressed in the *vsf-1* mutant while elevated in the overexpressing lines. These genes have independently been shown to positively regulate fruit firmness, for example, firmness is increased by overexpressing a *Nicotiana tabacum* XTH homolog [21], or reduced by knocking down the expression of *TBG6* [18] or *PMEU1* [11, 33]. This strongly supports the notion that the *VSF-1* regulatory network functions in primary cell wall remodeling of the expanding pericarp. Furthermore, we validated the potentially direct transcriptional activation of *TBG6*, a gene previously reported as a positive regulator of fruit firmness at the IMG stage [18]. However, *tbg6* mutant fruit exhibited only a relatively moderate reduction in firmness compared to *vsf-1* mutants, suggesting that VSF-1-mediated regulation of fruit firmness involves a broader suite of cell wall-modifying enzymes. This includes other direct or indirect targets such as *PMEU1,* which promotes pectin demethylesterification and subsequent cross-linking to increase cell wall stiffness [11], and *XTH* family members involved in modulating xyloglucan turnover [45]. Consequently, the pronounced firmness phenotype in *vsf-1* mutants is likely the result of the combined disruption of multiple pathways, including galactose metabolism, pectin modification, and hemicellulose remodeling, rather than the loss of *TBG6* alone.

These data reveal an important transcriptional module where VSF-1 activity maintains fruit structural integrity primarily at early fruit developmental stages. This finding also highlights the functional diversification of this bZIP subfamily I in climacteric fruit development, contrasting with the known developmental roles of its *Arabidopsis* orthologs, or the function of VSF-1 in response to hypo-osmotic stress [36, 39].

### Spatial regulation of cell wall architecture by VSF-1

Our TEM analysis revealed that VSF-1 plays a distinct, cell layer-specific role in modulating cell wall architecture within the tomato pericarp, depending on the developmental state of the parenchyma cells. This spatial divergence is directly linked to whether the cells are newly formed through division after anthesis (subepidermal region) or predetermined before anthesis (central region) [54].

The parenchyma cells adjacent to the epidermis originate from frequent cell divisions that continue after anthesis [54, 55]. In this region, *VSF-1* KO leads to thinner cell walls exclusively in these newly formed cells. Although DAP-seq analyses suggest that VSF-1 binds to the promoters of genes enriched in the ‘mitotic cell cycle process’, the lack of change in the total number of cell layers in the KO indicates that the primary function of VSF-1 is likely postmitotic: facilitating the primary assembly and reinforcement of the nascent cell wall. The integrity of these newly formed walls is thus critically dependent on the VSF-1 pathway.

In contrast, the central parenchyma cells largely lack mitotic activity after anthesis [54, 55]. Here, *VSF-1* overexpression induces pronounced wall thickening. Our quantitative TEM analyses revealed that central parenchyma cells possess comparatively thinner cell walls at baseline than do the subepidermal parenchyma cells. This structural gradient endows the central cells with a greater thickening potential and plasticity, rendering them more susceptible to the perturbations induced by *VSF-1* overexpression. Conversely, the thicker walls of the subepidermal parenchyma cells may have a limited capacity for further thickening, explaining their lack of a visible response in the overexpression background.

In summary, we posit a model wherein VSF-1 is indispensable for primary cell wall construction following division, but its potential to promote wall thickening is only fully realized in cells that exhibit high plasticity—a property intrinsically linked to a thinner cell wall baseline and the high biosynthetic capacity [56]. These findings underscore the fact that the resulting output of a genetic perturbation is profoundly shaped by the developmental and metabolic context of the specific cell population.

### A robust regulation of fruit firmness

Our phenotypic analysis revealed a functional divergence between gain-of-function and loss-of-function lines [57, 58]: overexpression of *VSF-1* led to pleiotropic effects, including enhanced fruit firmness, increased cell layers, and an elevated water loss rate. In contrast, the KO phenotype was limited primarily to reduced firmness, with major developmental and ripening traits unaffected. Consistently, far more genes exhibited altered expression in *VSF-1*-OE lines than in *vsf-1* mutants. This broader phenotypic spectrum and the greater number of DEGs in the overexpressing line versus the KO mutant suggest that functional redundancy [59], possibly involving other bZIP family members, may compensate for the loss of *VSF-1* in the mutant background. This underscores the existence of a robust, compensatory gene regulatory network dedicated to maintaining structural integrity during fruit development [58, 60]. By identifying *VSF-1* as a critical node governing cell wall properties and histological structure, our work provides new insights into the hierarchical organization of quality traits in fleshy fruits and underscores the multigene regulation of fruit texture [7].

Our findings also support the notion that fruit texture regulation occurs before ripening initiation through the coordinated deposition and configuration of wall polymers, which define the baseline firmness of the fruit [16, 61, 62]. The *VSF-1* mutant phenotype, which exhibits altered wall architecture and reduced firmness at the preripening stage, provides direct genetic evidence that perturbations in this early developmental window directly affect final fruit quality.

The upregulation of cuticle-associated genes in *VSF-1*-OE fruits coincided with the increased cuticular invagination. However, *VSF-1*-OE fruits exhibited higher postharvest water loss and enhanced toluidine blue permeability. It is plausible that increased invagination affects cuticle integrity, potentially creating additional pathways for water and dye diffusion. This is supported by recent evidence indicating that cuticular water loss is largely governed by structural features, such as polar pores, rather than bulk cutin content [63]. Thus, while *VSF-1* overexpression promotes the expression of cutin biosynthesis genes, it may simultaneously alter cuticle architecture in a manner that increases permeability. Furthermore, as water loss is a detrimental trait for postharvest fruit quality [63, 64], to circumvent these adverse effects on cuticle integrity and plant height utilizing fruit-specific promoters such as *PPC2*, which drives expression mainly in expanding mesocarp cells [65], offers a strategic approach to enhance fruit firmness while maintaining the functional properties of the fruit barrier and overall plant vigor.

### A negative feedback loop between *LOB1* and *VSF-1* in fruit texture control

Previous work identified *LOB1* as a master regulator promoting fruit softening during ripening in tomato. The expression of *VSF-1* was found to be highly repressed in *LOB1* knock-down lines, and activated in overexpression lines [32], initially suggesting the involvement of VSF-1 in texture regulation. Strikingly, our data indicate that VSF-1 functions as a positive regulator of firmness, opposing the known function of LOB1. This leads to the hypothesis that VSF-1 and LOB1 form a negative feedback loop that regulates fruit firmness.

The genes targeted by LOB1 are predominantly associated with cell wall loosening and degradation, including *EXP1*, *CEL2,* and the crucial pectate lyase *PL* [32]. However, our transcriptomic data reveal that overexpression of *VSF-1* significantly inhibits the transcript levels of *LOB1*. Furthermore, the expression of key cell wall-degrading genes directly activated by LOB1, such as *EXP1* [15, 22]*, CEL2* [15, 19], and *PL* [12], was strongly suppressed in the *VSF-1* overexpressing lines, with transcript levels reduced to 26%, 7%, and 30% of WT, respectively. Given that *PL* is documented as one of the most potent facilitators of fruit softening and that the double KO of *EXP1* and *CEL2* significantly enhances fruit firmness [12, 15], the severe downregulation of these three genes explains a substantial part of the pronounced firmness increase observed in *VSF-1* overexpressing fruit. However, DAP-seq analysis detected no VSF-1 binding enrichment at the *LOB1* promoter, indicating that VSF-1 does not directly repress *LOB1* transcription. This finding supports a model in which VSF-1 represses *LOB1* through an indirect mechanism. These data suggest that a balanced fruit texture—hard during fruit growth but soft for fruit ripening—is necessary and mediated, at least partially, by the indirect negative feedback of the VSF-1 and LOB1 TFs.

## Conclusion

This study functionally characterized the bZIP TF VSF-1. *vsf-1* mutants produced softer fruit, while *VSF-1*-OE lines generated firmer fruit compared to WT at identical stages, particularly during early development. Transcriptomic analysis revealed that VSF-1 is involved in cell wall dynamics, which is consistent with the reduced cell wall thickness and CWM content of *vsf-1* fruit, and increased cell wall thickness and CWM content of *VSF-1*-OE fruit. Combined with DAP-seq, *TBG6* was identified as a direct target, and we demonstrated that VSF-1 binds to and activates the *TBG6* promoter. Moreover, *tbg6* mutants displayed softer texture from the IMG to BR stages, which partially explains the reduction in firmness observed in *vsf-1* fruit. Collectively, this work reveals a novel role for a bZIP TF in regulating fruit firmness during development, a previously overlooked regulatory phase. It provides the first functional evidence for bZIP subfamily I factors in tomato firmness and identifies a direct target for improving fruit texture quality.

## Material and methods

### Plant materials and growth conditions

Tomato (*S. lycopersicum*) cultivar AC was used as the WT background for all genetic transformation and subsequent analyses. Seeds of both the WT and transgenic lines were surface-sterilized with 0.1% potassium permanganate for 20 min, followed by rinsing with deionized water. Seeds were germinated in a shaker at 160 rpm and 28°C until radicle emergence before transplantation into commercial potting mix. Seedlings were maintained in a greenhouse at the Zijingang Campus of Zhejiang University under controlled conditions: 70% relative humidity, with a 16-h light/8-h dark photoperiod. Flowers were tagged at anthesis (0 days postanthesis, 0 DPA), and fruits were harvested at key developmental stages, including immature green (IMG, 25 DPA), mature green (MG, 39 DPA), breaker (BR, onset of yellowish coloration), B3 (3 days after BR), and B7 (7 days after BR).

### Constructs and tomato transformation

For CRISPR/Cas9-mediated gene editing of *VSF-1* and *TBG6*, single guide RNAs (sgRNAs) were designed using the online tool SCORE (http://crispr.hzau.edu.cn/cgi-bin/crispr2/SCORE). The selected sgRNA sequences were cloned into the CDC45–1300 vector. The resulting constructs were transformed into tomato plants via *Agrobacterium*-mediated transformation. Mutations at the target site were validated by PCR amplification followed by Sanger sequencing. For the overexpression construct, the full-length coding sequence (CDS) of *VSF-1* was amplified and inserted into the 35S:pBTEX vector. The plasmid was transformed into *Agrobacterium tumefaciens* strain GV3101 for stable genetic transformation to generate *VSF-1* overexpression lines.

### Analysis of the fruit and plant phenotypes

Fruit firmness was assessed using a TA-XT plus texture analyzer (Stable Micro Systems, UK) as previously described [15]. Two types of measurements were performed on each fruit: whole-fruit compression using a P100 plate (100 mm in diameter) and pericarp penetration using a P2 probe (2 mm in diameter). A minimum of nine individual fruits per group were tested, and firmness was calculated based on the maximum compression force recorded (in Newtons).

Ethylene production was measured in B3 fruit, following a previously described method [32]. Fruits were first kept at room temperature for 4 h, then sealed individually in 300-ml containers for 2 h. A 1-ml sample of headspace gas was extracted from each container and injected into a 7890B gas chromatography system (Agilent Technologies, USA) for ethylene quantification. Ethylene concentrations were calculated from chromatographic peak areas, analyzing a minimum of 12 fruits per line.

Fruit weight was determined using 20 red ripe fruits per line. Total soluble solids content was measured using a handheld refractometer (ATAGO PAL-3, Japan). Juice was extracted from the apical portion of B7 fruit from a minimum of nine individual plants per line. Plant height was measured using a standard measuring tape, assessing a minimum of nine individual plants per line.

### Histological analysis of pericarp structure

Paraffin-embedded sections were prepared from the equatorial pericarp of MG tomato fruit following an established protocol [66]. Briefly, tissues were fixed in formalin-aceto-alcohol fixative for at least 48 h at room temperature, followed by standard processing including dehydration, clearing, paraffin infiltration, embedding, and sectioning at a thickness of 5 μm. Sections were stained with 0.5% (w/v) toluidine blue for 3 min for histological examination.

Morphometric measurements were conducted on the stained sections. Pericarp thickness was measured at three random regions for each of the six biological replicates per line. The number of cell layers was determined by counting cells along a transect perpendicular to the cuticle from the exocarp to the endocarp, with three random regions evaluated per biological replicate.

The cell density beneath the cuticle was quantified by manually counting cells (≥50% of their area within the region) within a 200 × 200 μm^2^ rectangular area positioned immediately below the cuticular layer. At least three random areas were assessed per biological replicate.

### TEM analysis

The ultrastructure of the pericarp from MG fruit was examined using TEM following a previously described method [67]. Ultrathin sections were examined using an HT-7820 TEM (Hitachi, Japan) operated at an accelerating voltage of 80 kV, and images of regions of interest were captured with a high-speed CMOS camera.

### RNA extraction, sequencing, and transcriptomic analysis

Total RNA was isolated from frozen tomato powder using a modified cetyltrimethylammonium bromide method [68]. RNA integrity and quality were assessed by Novogene (Beijing, China). Library preparation involved mRNA enrichment using oligo(dT) magnetic beads, followed by fragmentation and cDNA library construction. Libraries were pooled and sequenced on the DNBSEQ-T7 platform. Gene expression levels were normalized and expressed as FPKM. Genes with a fold change >1.50 or < 0.67 and an FDR ≤ 0.05 were defined as DEGs. GO enrichment analysis was conducted using the tools available at the GO website (https://geneontology.org/).

### RT-qPCR analysis

Total RNA (1 μg) was reverse-transcribed into cDNA using HiScript III RT SuperMix (Vazyme, China) according to the manufacturer’s instructions, incorporating a gDNA wiper step to eliminate genomic DNA contamination. The synthesized cDNA was diluted 5-fold, and 2 μl of the dilution was used as the template for reverse transcription quantitative polymerase chain reaction (RT-qPCR). Reactions were performed on a CFX96 Touch Real-Time PCR Detection System (Bio-Rad, USA) using ChamQ SYBR qPCR Master Mix (Vazyme). The tomato *SlEF1α* gene (Solyc06g005060) served as the internal reference for normalization. Relative expression levels of target genes were calculated using the 2^(−ΔΔCt) method.

### DAP-seq and bioinformatic analysis

DAP-Seq was performed according to the protocol described by Bartlett et al. with minor modifications [69]. Genomic DNA was extracted from tomato fruit tissues and fragmented to construct an affinity-purified library. The VSF-1 protein was expressed *in vitro* using a cell-free system. Libraries from two independent biological replicates and an input control were subjected to high-throughput sequencing on a NovaSeq platform, generating 150 bp paired-end raw reads. Raw data were quality-filtered using fastp [70]. Recurrent DNA motifs within the peak regions were identified using MEME-chip [49]. Genomic DNA extraction, library construction, sequencing, and primary data analysis were performed by Yongji Biotechnology Co., Ltd. (Guangzhou, China). GO enrichment analysis was conducted using the tools available at the GO website (https://geneontology.org/).

### Subcellular localization

The full-length CDS of *VSF-1* was cloned into the pCAMBIA1300-35S-EGFP vector. The resulting plasmid was transformed into *A. tumefaciens* GV3101::pSoup. Bacterial cultures were resuspended in an infiltration buffer (10 mM MES, 10 mM MgCl₂, and 150 μM acetosyringone, pH 5.6) to an OD600 of 0.70. Suspensions containing the VSF-1-EGFP construct or the empty vector (control) were individually infiltrated into leaves of *N. benthamiana* plants stably expressing a nuclear-localized red fluorescent protein. After 48 h, subcellular localization was observed using a confocal laser scanning microscope (Nikon A1-SHS, Japan) with a 488-nm excitation laser and a 520–560 nm emission filter.

### Yeast one-hybrid assay

The promoter sequence of *TBG6* was cloned into the pAbAi vector and transformed into the Y1HGold yeast strain. The full-length CDS of *VSF-1* was inserted into the pGADT7-AD vector. The recombinant plasmid, along with the empty pGADT7-AD vector (as a negative control), was then individually introduced into the Y1HGold yeast strain harboring the *TBG6*-pAbAi bait. Transformants were selected on SD/-Leu medium containing 0 to 400 ng ml^−1^ aureobasidin A, and incubated at 28°C for 3 days to assess protein–DNA interactions.

### Electrophoretic mobility shift assays

The full-length cDNA of *VSF-1* was cloned into the pGEX-4 T-1 vector and transformed into *Escherichia coli* Rosetta(DE3)pLysS for the expression of a recombinant VSF-1-GST fusion protein. Protein expression was induced with 0.5 mM IPTG at 16°C for 20 h, and the recombinant protein was purified using glutathione Sepharose beads (GE Healthcare, Chicago, IL, USA) according to the manufacturer’s instructions. EMSAs were performed using a Chemiluminescent EMSA Kit (Beyotime Biotechnology, Shanghai, China).

### Dual-luciferase reporter assay

Transient expression assays for the dual-LUC assay were performed as previously described [71]. The full-length CDS of *VSF-1* was cloned into the pGreenII 0029 62-SK vector (effector). The target promoter fragment was inserted into the pGreenII 0800-LUC vector (reporter), containing the firefly LUC and Renilla LUC (REN) reporter genes. Agrobacterial suspensions containing the TF (effector) and the promoter (reporter) were mixed at a 10:1 (v/v) ratio and co-infiltrated into leaves of *N. benthamiana*. The empty pGreenII 0029 62-SK vector served as the negative control, with its relative LUC/REN ratio normalized to 1. Three days postinfiltration, LUC and REN activities were measured using the Dual-Luciferase® Reporter Assay System (Promega, USA). Each combination was tested with at least three independent biological replicates. For the synthetic *VBM* promoter, it contained nine tandem repeats of the TF binding site (derived from the VSF-1 *Arabidopsis* homolog motif in the JASPAR database) [43], positioned upstream of a mini35S core promoter.

### Extraction of CWM

CWM extraction was performed with modifications to a previously described method [72]. Approximately, 10 g of frozen fruit pulp was homogenized in 40 ml of 95% (v/v) ethanol, vortexed, and heated in a boiling water bath for 30 min. The insoluble residue was sequentially washed with 80% ethanol, a chloroform:methanol (1:1, v/v) solution, and acetone. The material was then air-dried in a fume hood for 12 h, followed by further drying in an oven at 40°C. Starch was removed from the resulting alcohol-insoluble residue by digestion with α-amylase [73]. The complete absence of starch was confirmed by iodine/potassium iodide staining prior to final sample weighing.

### Water loss measurement and toluidine blue staining

Water loss was measured using a minimum of eight individual fruits at the B3 stage. After initial weighing, the fruits were placed in an undisturbed environment at 26°C. The weight was measured every 10 days to calculate the water loss rate. Toluidine blue staining was performed on WT and transgenic fruit at the MG stage, following an established protocol with modifications [74]. Freshly harvested fruits were immersed in 1% toluidine blue solution for 12 h, rinsed with distilled water, and then images were captured to document the staining level of the fruit surface.

### Statistical analysis and visualization

Statistical analyses were performed using a two-tailed Student’s *t-*test to determine significant differences between groups. Significance levels are denoted as: ^*^P < 0.05, ^**^P < 0.01, and ^***^P < 0.001. Data processing was conducted using Microsoft Excel 2021. Graphs were generated with GraphPad Prism 8 and Microsoft Excel 2021. The gene structure schematic was generated using the online tool Exon-Intron Graphic Maker (http://www.wormweb.org/exonintron).

## Supplementary Material

Web_Material_uhag181

## Data Availability

Raw RNA-seq reads (accession number CRA035235) and DAP-seq data (accession number CRA035249) are available at the Genome Sequence Archive (https://ngdc.cncb.ac.cn/gsa) in National Genomics Data Center, Beijing Institute of Genomics, China National Center for Bioinformation, Chinese Academy of Sciences.
