# Supplementary material for: The novel function of the bZIP transcription factor VSF-1 in mediating fruit firmness in tomato
Source: Hortic Res. 2026 Apr 30;13(10):uhag181. doi: 10.1093/hr/uhag181 (PMC13600432; doi:10.1093/hr/uhag181)
Supplement: Supplementary file 1 [file web_material_uhag181.zip › Fig. S1-S7.pdf]

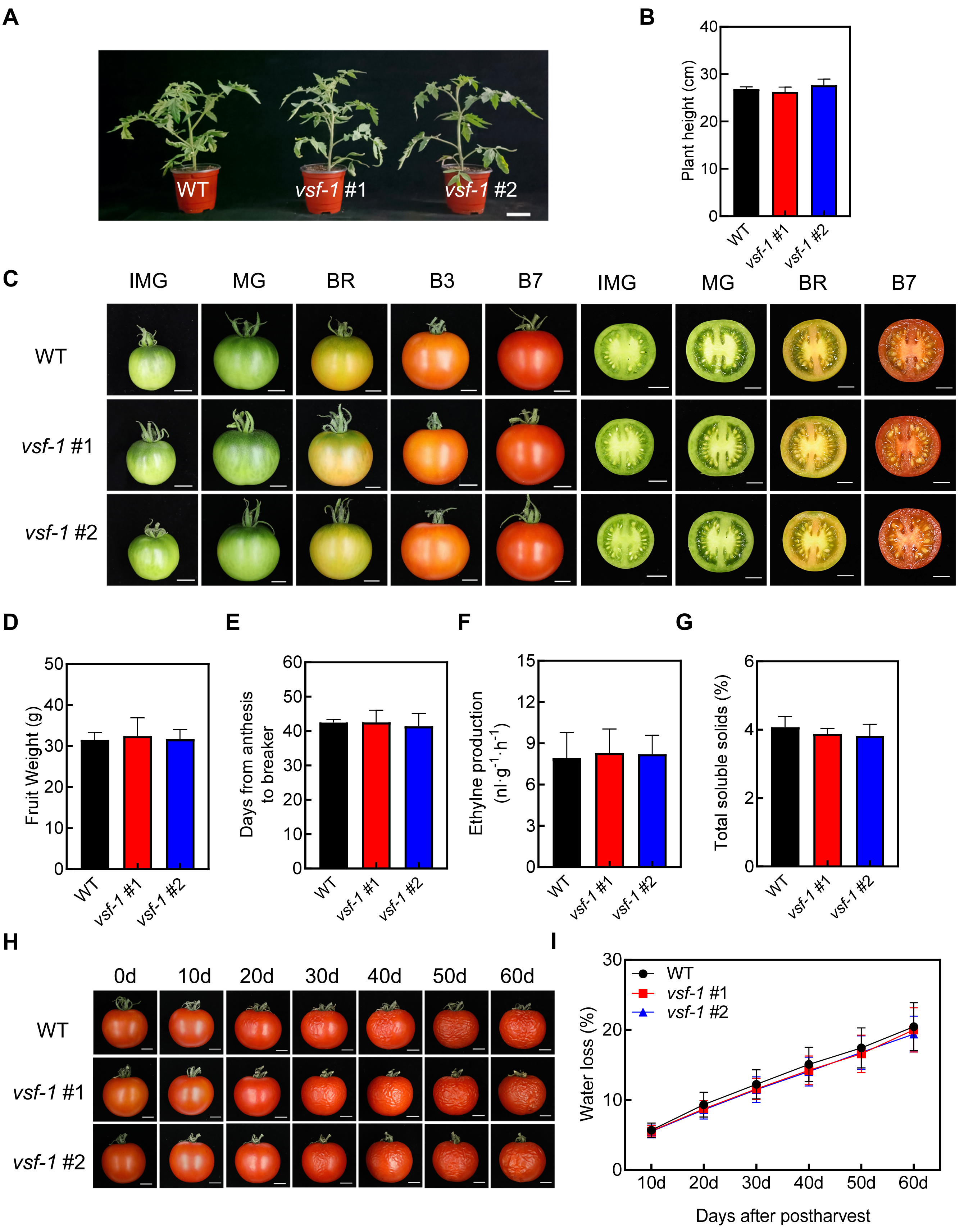

Figure S1. Phenotypic comparison of the *vsf-1* mutant and WT plants, showing comparable growth phenotypes, fruit ripening process and postharvest shelf life. **(A)** Representative images of whole WT and *vsf-1* mutants at 4 weeks after germination. The scale bar represents 5 cm. **(B)** Corresponding statistical analysis of plant height for WT and *vsf-1* mutant plants. Data are presented as means ± SD ($n = 9$). **(C-G)** *vsf-1* mutant fruit showed no ripening alteration compared to WT, including fruit appearance during fruit ripening **(C)**, fruit fresh weight **(D)**, days from anthesis to breaker stage **(E)**, ethylene production **(F)** and total soluble solids content **(G)**. The scale bar represents 1 cm. Data are presented as means ± SD ($n \geq 9$). **(H)** Postharvest phenotyping of WT and *vsf-1* mutant fruit harvested at B3 stage. Fruits were stored at 26 °C and imaged at the indicated days (0, 10, 20, 30, 40, 50 and 60 days) after harvest. The scale bar represents 1 cm. **(I)** Corresponding statistical analysis of fruit water loss rate. Data are presented as means ± SD ($n \geq 9$).

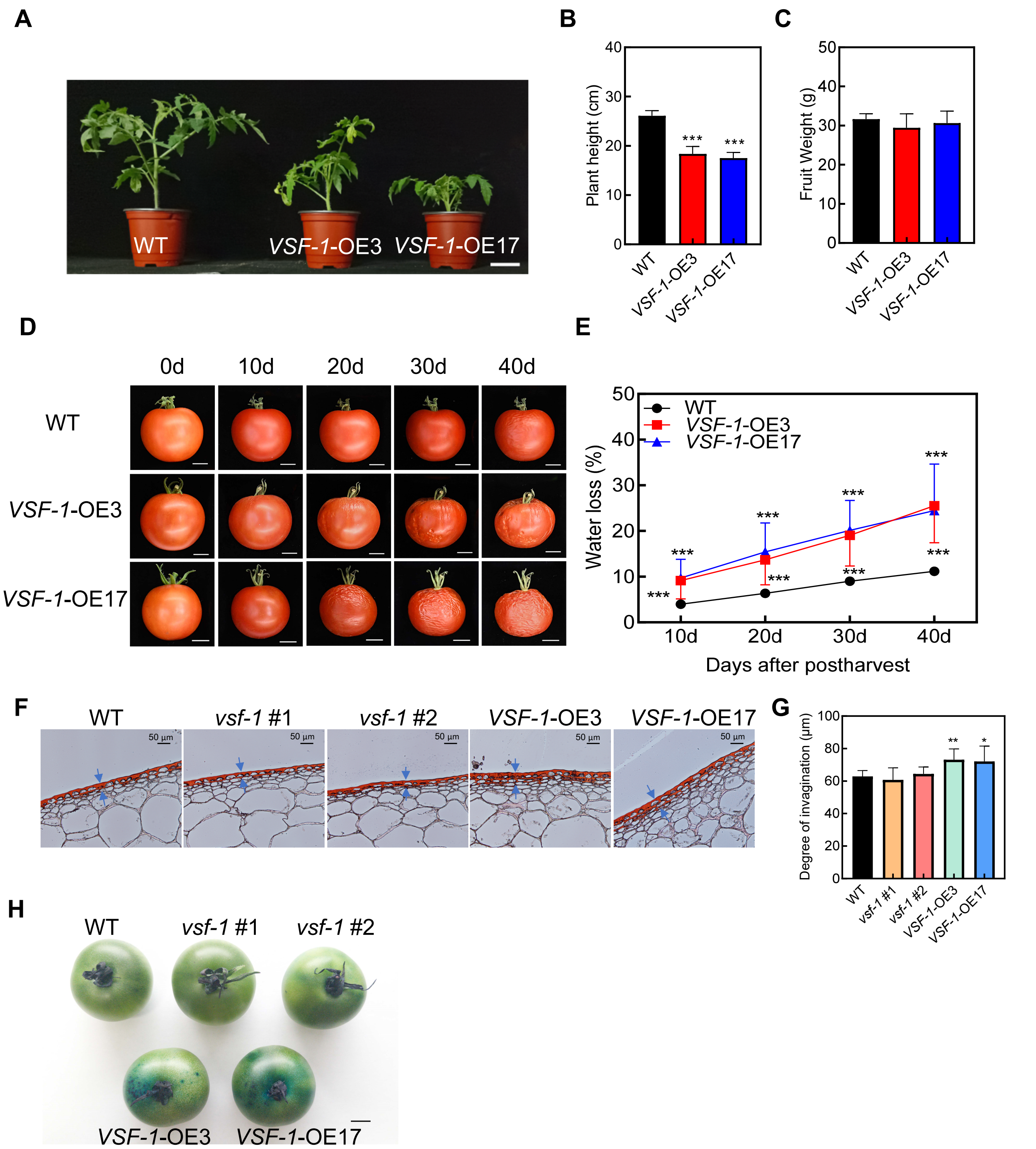

Figure S2. Overexpression of *VSF-1* leads to decreased plant height and increased water loss in tomato fruits. **(A)** Representative images of whole WT and *VSF-1*-OE plants at 4 weeks after germination. The scale bar represents 5 cm. **(B)** Corresponding statistical analysis of plant height. Data are presented as means ± SD ($n = 16$). **(C)** Fruit fresh weight in WT and *VSF-1* overexpression lines (*VSF-1*-OE3, *VSF-1*-OE17). Data are presented as means ± SD ($n = 20$). **(D)** Postharvest phenotyping of WT, *VSF-1*-OE3 and *VSF-1*-OE17 fruit harvested at B3 stage. Fruits were stored at 26 °C and imaged at the indicated days (0, 10, 20, 30 and 40 days) after harvest. The scale bar represents 1 cm. **(E)** Corresponding statistical analysis of fruit water loss rate. Data are presented as means ± SD ($n \geq 8$). ***$P < 0.001$. **(F)** Cuticular invagination in WT and *VSF-1*-OE fruits visualized by Oil Red O staining. The scale bar represents 50 µm**. (G)** Statistical analysis of cuticular invagination. Data are presented as means ± SD (n = 6). **(H)** Permeability to toluidine blue dye in MG fruit after 12 hours incubation. The scale bar represents 1 cm.

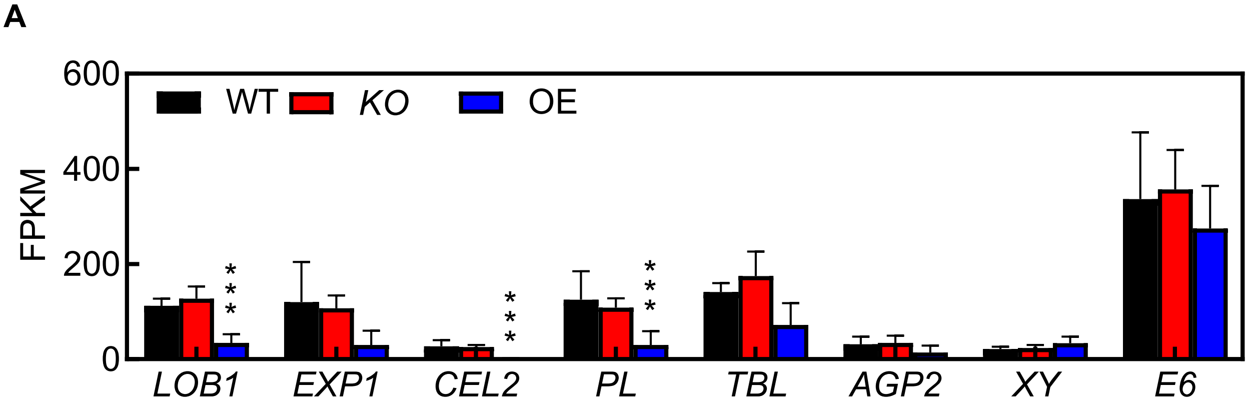

Figure S3. RNA-seq analysis of the gene expression of selected *LOB1*-associated cell wall genes in MG stage fruit from WT, *vsf-1* and *VSF-1*-OE lines. Data are presented as means ± SD ($n = 3$).

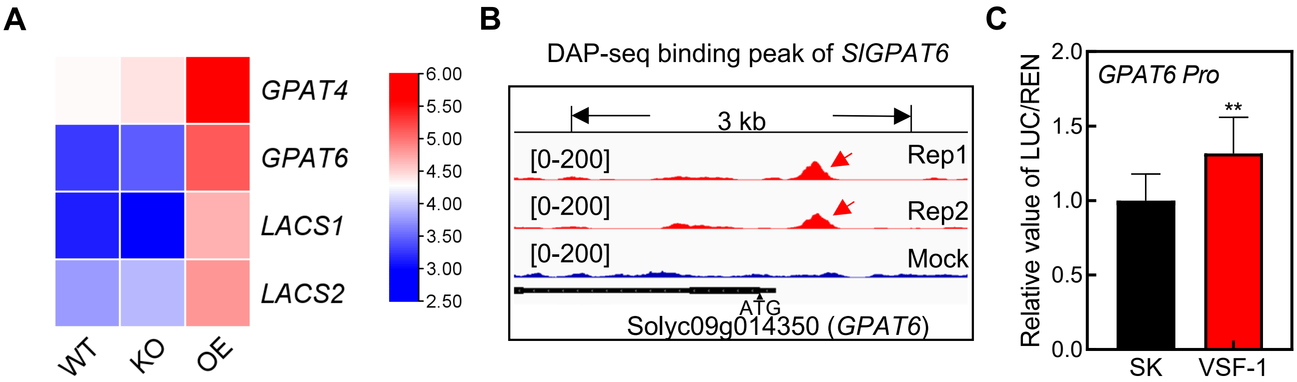

Figure S4. VSF-1 targets the *GPAT6* promoter and activates it in a dual luciferase reporter assay. **(A)** Heatmap showing FPKM values of four cuticle-related genes from the "Cutin-based cuticle development" GO term in WT, KO, and OE fruits. **(B)** VSF-1 binding peaks present in the promoter region of *GPAT6* as determined by DAP-seq. Red arrows indicate the high-confidence binding peak in the promoter region. **(C)** Dual-luciferase transactivation assay in tobacco leaves. VSF-1 significantly enhanced the activity of the *GPAT6* promoter-driven luciferase reporter relative to the empty vector control. The relative LUC activities normalized to the REN activity are shown (LUC/REN). SK (empty vector) serves as the negative control. Data are presented as means ± SD ($n = 3$).

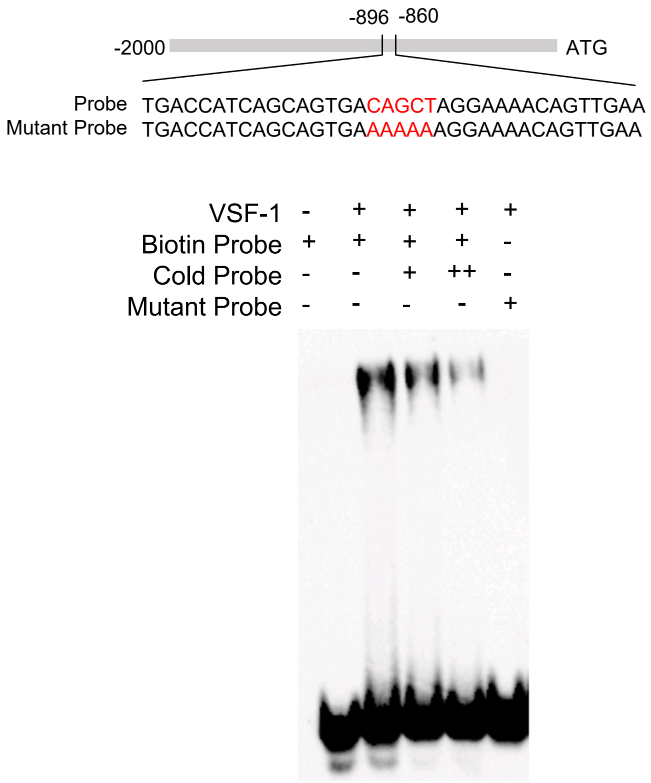

Figure S5. EMSA data demonstrate the binding of VSF-1 to the *TBG6* promoter..

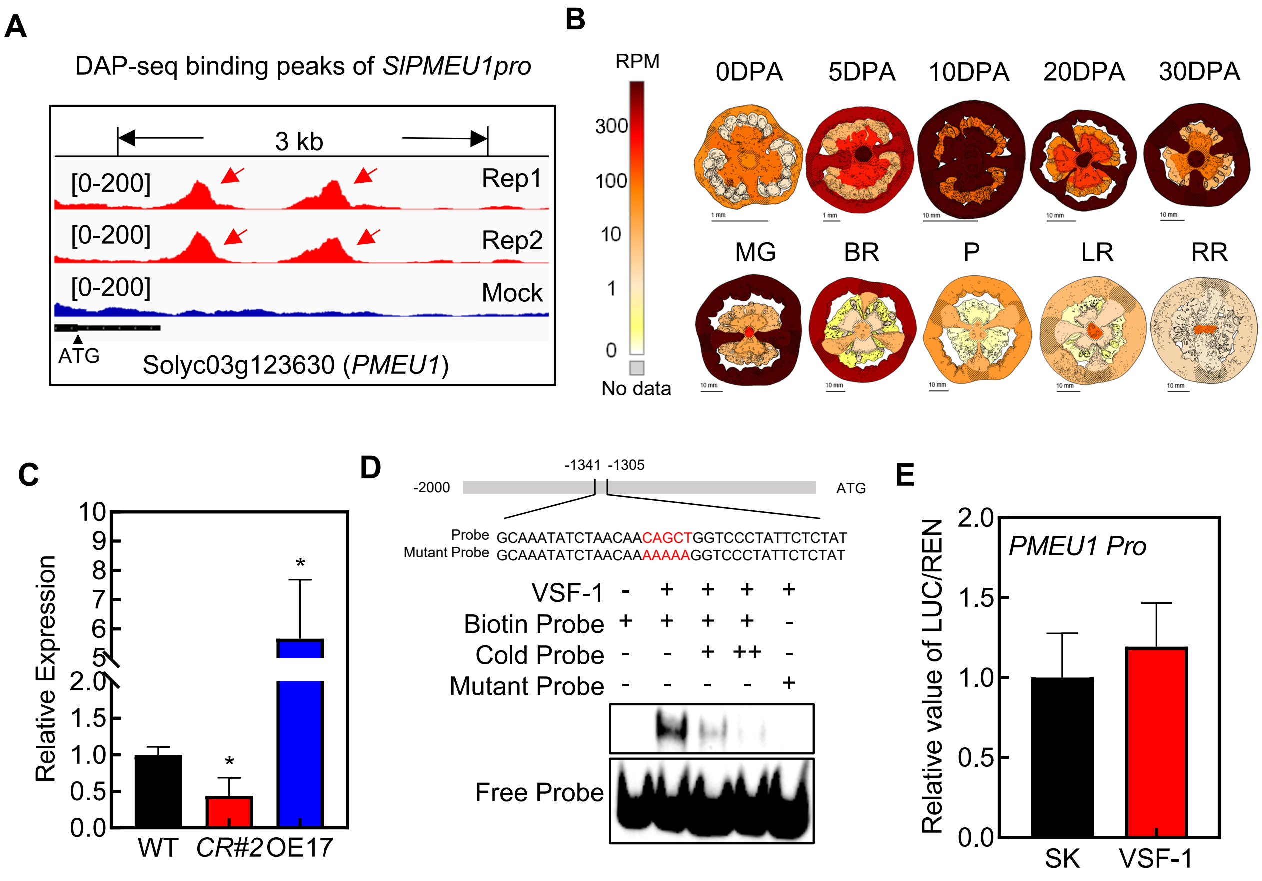

Figure S6. VSF-1 activates *PMEU1* transcription. **(A)** VSF-1 binding peaks present in the *PMEU1* promoter region as determined by DAP-seq. Red arrows indicate the high-confidence binding peaks in the promoter region. **(B)** Expression pattern of the *PMEU1* gene during fruit development. Expression profile from TEA database[41,42] (https://tea.solgenomics.net/). P, pink; LR, light red; RR, red ripe; RPM, reads per million mapped reads. **(C)** Relative transcript abundance of *PMEU1* in *vsf-1* and *VSF-1*-OE lines, quantified by qRT-PCR. Data are expressed as fold change versus WT expression and normalized to the reference gene *EF1α*. $^{*}P < 0.05$. Data are presented as means ± SD ($n = 3$). **(D)** EMSA results show the direct binding of VSF-1 to the *PMEU1* promoter. Symbols (+), (−), and (++) indicate the presence, absence, or increasing concentrations of the respective components. The full gel image is provided in Fig. S7.**(E)** Transient transcriptional activity assay show VSF-1 failed to activate the *PMEU1* promoter. The relative LUC activities normalized to the REN activity are shown (LUC/REN). SK (empty vector) serves as the negative control. Data are presented as means ± SD ($n = 4$).

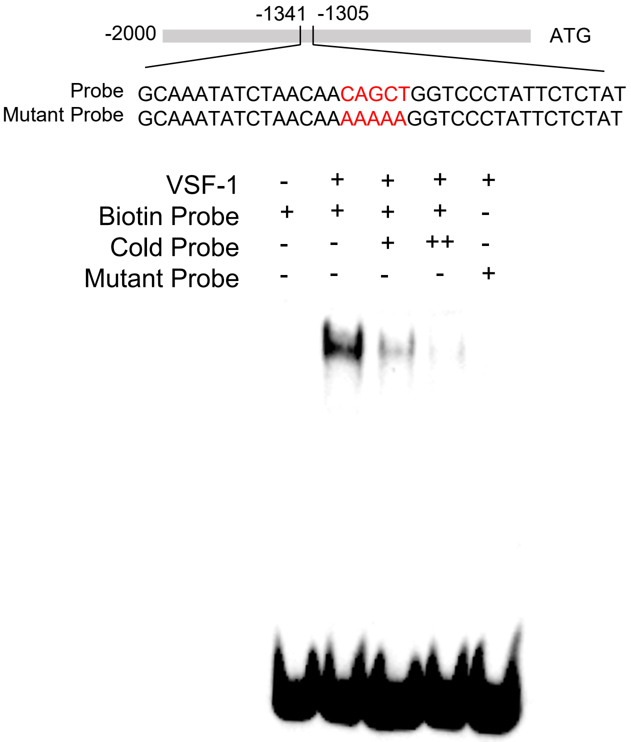

Figure S7. EMSA data demonstrate the binding of VSF-1 to the *PMEU1* promoter.
