# Supplementary material for: The novel function of the bZIP transcription factor VSF-1 in mediating fruit firmness in tomato
Source: Hortic Res. 2026 Apr 30;13(10):uhag181. doi: 10.1093/hr/uhag181 (PMC13600432; doi:10.1093/hr/uhag181)
Supplement: Supplementary file 1 [file web_material_uhag181.zip › Table S2.pdf]

Table S2. Candidate bZIP transcription factors as potential interacting partners of VSF-1

Gene ID	Annotation	WT (FPKM)	Pearson
Solyc01g108080	ABSCISIC ACID-INSENSITIVE 5-like protein 7	47.00	0.77
Solyc04g078840	AREB	12.04	0.76
Solyc04g080740	BZIP transcription factor	85.94	0.74
Solyc01g110480	Basic-leucine zipper (BZIP) transcription factor family protein	19.83	0.71
Solyc08g022080	BZIP transcription factor	78.43	0.71
Solyc02g062950	G-box binding	24.19	0.69
Solyc01g104650	ABSCISIC ACID-INSENSITIVE 5-like protein 5	50.59	0.66
Solyc01g008980	Abscisic acid-insensitive 5-like protein	10.67	0.66
Solyc04g071160	Basic-leucine zipper (BZIP) transcription factor family protein	51.04	0.66
