# Supplementary material for: The novel function of the bZIP transcription factor VSF-1 in mediating fruit firmness in tomato
Source: Hortic Res. 2026 Apr 30;13(10):uhag181. doi: 10.1093/hr/uhag181 (PMC13600432; doi:10.1093/hr/uhag181)
Supplement: Supplementary file 1 [file web_material_uhag181.zip › Table_S1.pdf]

Table S1. Cell wall-related DEGs in the *VSF-1* overexpression line compared to the WT

Gene ID	Annotation	KO fold	OE fold	Blast hit in *Arabidopsis*	Function
**Cellulose**					
Solyc09g072820.4	Cellulose synthase	0.95	12.42	AT5G44030, CesA4	Involved in secondary cell wall biosynthesis
Solyc04g071650.4	Cellulose synthase	0.80	6.09	AT5G64740, CesA6	Related to primary cell wall biosynthesis
Solyc12g056580.2	Cellulose synthase	0.83	2.89	AT2G21770, CesA9	Related to primary cell wall biosynthesis
Solyc08g061100.3	Cellulose synthase	0.82	2.41	AT4G32410, CesA1	Related to primary cell wall biosynthesis
Solyc01g087210.3	Cellulose synthase	0.87	2.37	AT5G05170, CesA3	Related to primary cell wall biosynthesis
Solyc11g005560.3	Cellulose synthase	0.85	2.25	AT5G64740, CesA6	Related to primary cell wall biosynthesis
Solyc02g065765.1	COBRA-like protein	0.79	3.57	AT5G60920, COBRA	Facilitate cellulose crystallization from the emerging β1–4-glucan chains
Solyc02g089120.4	COBRA-like protein	1.30	0.45	AT5G60920, COBRA	Facilitate cellulose crystallization from the emerging β1–4-glucan chains
Solyc02g089130.4	COBRA-like protein	1.25	0.39	AT5G15630, COBL4	Involved in secondary cell wall biosynthesis
Solyc04g078750.3	Late embryogenesis abundant protein	0.50	2.98	AT1G45688, CC1	Interact with the cellulose synthase complex and microtubules
Solyc03g113680.4	Microtubule-associated protein TORTIFOLIA1	1.08	2.79	AT4G27060, TOR1	Play a role in control of microtubule dependent anisotropic cell elongation
Solyc04g008040.4	Microtubule-associated protein 70	0.91	5.50	AT1G68060, MAP70-1	Interact with microtubules
Solyc03g116370.3	Microtubule-associated protein RP/EB family member 1C	0.84	4.88	AT5G62500, EB1B	Participate in microtubule bundle formation or maintenance
Solyc01g102580.3	Endo-1,4-beta-glucanase (*Cel3*)	0.74	2.18	AT5G49720, GH9A1	Endo-1,4-beta-D-glucanase, involved in cellulose biosynthesis
**Hemicellulose**					
Solyc05g054590.4	UDP-glucuronic acid decarboxylase	0.85	6.42	AT2G28760, UXS6	Participate in xylan synthesis
Solyc02g092840.1	Xyloglucan galactosyltransferase KATAMARI1	0.87	2.28	AT5G62220, GT18	Involved in galactosyl-substitution of xyloglucan at position 2
Solyc09g064470.3	Xyloglucan galactosyltransferase KATAMARI1	0.97	3.32	AT2G20370, MUR3	Involved in the biosynthesis of fucose
Solyc04g072850.4	Beta-D-xylosidase	0.65	5.83	AT5G09730, AtXYL3	Participate in the in-vivo hydrolysis of arabinan
Solyc10g047030.3	LEXYL1	0.51	0.13	AT5G64570, XYL4	Modify cell wall polysaccharides
Solyc04g007160.1	Alpha-xylosidase 1-like	0.97	0.49	AT1G68560, XYL1	Degrade XyG by trimming side chains
Solyc08g006310.3	Cellulose synthase-like C4, glycosyltransferase family 2	0.98	3.09	AT4G31590, CSLC5	A XyG glucan synthase
Solyc01g067930.4	Xyloglucan 6-xylosyltransferase 1	1.13	2.03	AT3G62720, XXT1	Add the xylosyl residue from UDP-xylose onto a glucan backbone chain

Solyc01g111340.4	Endo-1,4-beta-xylanase	0.85	0.21	AT1G58370, XYN1	A protein with xylanase activity
Solyc10g083670.3	Glucomannan 4-beta-mannosyltransferase 2	0.98	6.18	AT5G03760, ATCSLA09	Synthesise glucomannan
Solyc06g074630.4	Glucomannan 4-beta-mannosyltransferase 2	0.82	8.55	AT5G22740, CSLA02	Synthesize galactoglucomannan
Solyc11g066820.2	Glucomannan 4-beta-mannosyltransferase 2	1.10	2.66	AT5G22740, CSLA02	Synthesize galactoglucomannan
Solyc03g019890.3	Beta-galactosidase 7	0.93	5.01	AT5G63810, BGAL10	Involved in the side chain trimming of xyloglucans (XyG), the major hemicellulose in cell walls
Solyc12g011023.1	Xyloglucan endotransglucosylase/hydrolase	0.40	27.71	AT3G23730, XTH16	Regulate cell wall dynamic remodeling
Solyc07g055990.3	Xyloglucan endotransglucosylase/hydrolase	0.35	13.07	AT3G23730, XTH16	Regulate cell wall dynamic remodeling
Solyc07g056000.2	Xyloglucan endotransglucosylase/hydrolase	1.10	11.03	AT4G14130, XTH15	Regulate cell wall dynamic remodeling
Solyc02g080160.4	Xyloglucan endotransglucosylase/hydrolase	3.39	10.00	AT1G11545, XTH8	Regulate cell wall dynamic remodeling
Solyc03g031800.3	Xyloglucan endotransglucosylase/hydrolase	0.60	9.81	AT1G11545, XTH8	Regulate cell wall dynamic remodeling
Solyc12g098603.1	Xyloglucan endotransglucosylase/hydrolase	0.17	5.97	AT4G14130, XTH15	Regulate cell wall dynamic remodeling
Solyc09g008320.4	Xyloglucan endotransglucosylase/hydrolase	0.61	5.80	AT2G36870, XTH32	Regulate cell wall dynamic remodeling
Solyc03g093080.3	Xyloglucan endotransglucosylase/hydrolase	0.46	5.20	AT4G25810, XTH23	Regulate cell wall dynamic remodeling
Solyc01g099630.4	Xyloglucan endotransglucosylase/hydrolase 1 (*XTH1*)	0.56	4.24	AT5G13870, XTH5	Regulate cell wall dynamic remodeling
Solyc04g008210.2	ETAG-A3 (*XTH8*)	0.50	3.14	AT2G01850, XTH27	Regulate cell wall dynamic remodeling
Solyc01g080010.2	Xyloglucan endoglucanase inhibitor	0.61	1.99	AT1G03220, SAP2	Homologous to carrot EDGP and tomato XEGIP
**Pectin**					
Solyc02g065530.4	Hexosyltransferase	0.55	39.47	AT3G50760, GATL2	Involved in cell wall biosynthesis
Solyc04g079860.1	Hexosyltransferase	1.08	3.75	AT3G50760, GATL2	Involved in cell wall biosynthesis
Solyc05g009820.4	Hexosyltransferase	0.47	2.71	AT1G13250, GATL3	Participate in pectic polysaccharide synthesis
Solyc09g075560.1	Hexosyltransferase	0.60	2.38	AT3G62660, GATL7	Protein with putative galacturonosyltransferase activity
Solyc12g010200.2	Hexosyltransferase	1.03	0.21	AT1G06780, GAUT6	Involved in pectin synthesis
Solyc07g062130.3	Trifunctional UDP-glucose 4,6-dehydratase/UDP-4-keto-6-deoxy-D-glucose 3,5-	1.01	3.05	AT1G78570, RHM1	A UDP-L-Rhamnose synthase involved in the biosynthesis of rhamnose
Solyc11g010600.2	O-fucosyltransferase	1.02	2.11	AT3G26370, PAGR	Affect the biosynthesis of RG-I arabinogalactans
Solyc03g019790.3	Alpha-galactosidase	0.80	4.51	AT5G08370, AGAL2	Involved in cell wall metabolism
Solyc02g082140.3	Galactan beta-1,4-galactosyltransferase GALS3	0.86	2.07	AT4G20170, GALS3	Add galactose onto existing galactose residues attached to the rhamnogalacturonan-I (RG-I) backbone.

Solyc02g084210.1	GDP-mannose 4,6-dehydratase	1.31	18.54	AT3G51160, MUR1	Catalyze the first step in the de novo synthesis of GDP-L-fucose
Solyc08g007310.3	Exostosin-like	1.12	2.21	AT5G33290, XGD1	Involved in pectin biosynthesis
Solyc01g111540.4	Beta-galactosidase	0.19	22.87	AT2G16730, BGAL13	Putative beta-galactosidase
Solyc06g062580.3	Beta-galactosidase	1.02	9.89	AT3G13750, BGAL1	Hydrolyze β-galactoside bonds
Solyc02g084720.3	Beta-galactosidase 6 (*TBG6*)	0.78	4.52	AT4G36360, BGAL3	Putative beta-galactosidase
Solyc11g018500.3	Beta-galactosidase	1.13	3.81	AT1G77410, BGAL16	Hydrolyze β-galactoside bonds
Solyc11g069270.2	Beta-galactosidase 5	0.76	2.07	AT2G28470, BGAL8	Putative beta-galactosidase
Solyc10g086480.3	Pectin acetylesterase	0.72	2.45	AT3G09410, PAE5	Regulate pectin acetylation
Solyc10g038130.3	Pectin acetylesterase	0.89	1.90	AT5G26670, PAE10	Regulate pectin acetylation
Solyc05g014000.4	Pectate lyase	0.58	7.89	AT4G24780, PLL19	Cleave pectin chains via elimination
Solyc09g061890.3	Pectate lyase	15.16	5.89	AT4G24780, PLL19	Cleave pectin chains via elimination
Solyc02g031960.3	Pectate lyase	0.40	4.03	AT1G14420, PLL1	Pectate lyase-like protein (PLL)
Solyc06g083580.4	Pectate lyase	0.85	3.30	AT4G24780, PLL19	Cleave pectin chains via elimination
Solyc03g071570.3	Pectate lyase	0.79	2.77	AT5G04310, PLL12	Pectin-modifying gene involved in guard cell wall modifications
Solyc03g111690.4	Pectate lyase (*PL*)	0.86	0.24	AT4G24780, PLL19	Cleave pectin chains via elimination
Solyc04g015530.3	Pectin lyase-like superfamily protein	1.17	0.14	AT3G57510, ADPG1	Polygalacturonase protein
Solyc06g009190.4	Pectinesterase	0.52	16.22	AT4G02300, PMEI-PME39	Pectin methylesterase inhibitor; INVI-PMEI protein isoform which clustered with a PME
Solyc03g123620.4	Pectinesterase	0.35	10.45	AT1G53840, PME1	Catalyze the demethylation of pectin
Solyc03g123630.4	Pectin methylesterase pmeu1 (*PMEU1*)	0.43	5.95	AT3G14310, PME3	Catalyze the demethylation of pectin
Solyc06g051960.3	Pectinesterase	0.38	4.07	AT3G49220, PME34	Pectin methylesterase involved in transpiration, heat tolerance
Solyc07g017600.3	Pectinesterase	0.98	2.74	AT4G33220, PMEI-PME44	Catalyze the demethylation of pectin
Solyc03g083360.3	Pectinesterase	0.71	2.46	AT5G53370, PMEI-PME61	Catalyze the demethylation of pectin
Solyc07g064170.3	Pectin methylesterase 1	1.09	0.51	AT1G11580, PMEI-PME18	Catalyze the demethylation of pectin
Solyc07g042390.3	Plant invertase/pectin methylesterase inhibitor superfamily protein	1.08	2.86	AT1G62770, PMEI9	Pectin methylesterase inhibitor
Solyc03g083770.1	Plant invertase/pectin methylesterase inhibitor superfamily protein, putative	0.83	0.19	AT5G62360, PMEI13	Pectin methylesterase inhibitor
**Cell wall proteins**					

Solyc07g054310.3	Cell wall protein	1.61	9.28	AT2G20870	Cell wall protein precursor
Solyc02g078850.1	Glycine-rich protein	0.24	14.60	AT4G21620	Extracellular glycine-rich structural protein
Solyc09g097760.3	Glycine-rich protein	0.62	0.09	AT2G05540	Extracellular glycine-rich structural protein
Solyc06g076220.3	Expansin18	1.04	6.72	AT2G39700, EXPA4	A putative expansin
Solyc04g081870.4	Expansin A11	7.76	12.85	AT1G20190, EXPA11	A putative expansin
Solyc11g005150.3	Extensin X55687	0.80	5.16	AT1G12040, LRX1	A chimeric leucine-rich repeat/extensin protein
Solyc10g005960.1	Fasciclin-like arabinogalactan protein	0.41	11.86	AT5G55730, FLA1	A subclass of arabinogalactan proteins (AGPs)
Solyc07g053540.1	Fasciclin-like arabinogalactan protein 9	0.69	9.50	AT1G03870, FLA9	A subclass of arabinogalactan proteins (AGPs)
Solyc12g015690.3	Fasciclin-like arabinogalactan protein 1	0.54	5.69	AT5G55730, FLA1	A subclass of arabinogalactan proteins (AGPs)
Solyc07g045440.1	Fasciclin-like arabinogalactan protein 2	0.53	5.64	AT4G12730, FLA2	A subclass of arabinogalactan proteins (AGPs)
Solyc10g081720.2	Fasciclin-like arabinogalactan protein 17	0.69	2.67	AT5G06390, FLA17	A subclass of arabinogalactan proteins (AGPs)
Solyc02g092790.3	Arabinogalactan	0.55	4.22	N/A	Arabinogalactan protein
Solyc05g007930.3	Beta-1,3-galactosyltransferase 15	1.24	5.05	AT1G26810, GALT1	Add β-1,3-Gal to the termini of N-glycans to form the Lewis a epitope on plant glycoproteins.
**Cell wall deposition**					
Solyc02g079490.3	HXXXD-type acyl-transferase family protein	0.73	0.07	AT5G48930, HCT	Involved in the lignin biosynthetic pathway
Solyc01g006360.4	Callose synthase 9	1.56	1.83	AT3G07160, GSL10	May be involved in the synthesis of the cell wall component callose
Solyc07g049370.2	Endo-1,3-β-glucanase	1.14	6.00	AT4G14080, MEE48	Involved in the formation of the pollen wall
Solyc12g014420.2	Endo-1,3-β-glucanase	1.34	3.24	AT4G14080, MEE48	Involved in the formation of the pollen wall
Solyc08g083310.3	Endo-1,3-β-glucanase	0.89	2.14	AT5G42100, BG_PPAP	Involved in plasmodesmal callose degradation
Solyc07g005330.3	Endo-1,3-β-glucanase	1.00	1.93	AT4G14080, MEE48	Involved in the formation of the pollen wall
Solyc01g008620.4	Endo-1,3-β-glucanase	0.21	0.04	AT3G57260, PR2	Beta 1,3-glucanase; callose degradation enzyme
**Cell wall signaling**					
Solyc10g006870.1	Receptor-like protein kinase THESEUS 1	0.65	3.02	AT5G54380, THE1	Encode THESEUS1 (THE1), a receptor kinase required for cell elongation during vegetative growth.
Solyc03g115710.1	Receptor-like protein kinase THESEUS 1	0.32	2.68	AT5G61350, CAP1	Influence vesicle trafficking and the cytoskeletal arrangement
Solyc01g096360.3	Rho GTPase-activating protein 3	0.93	2.86	AT2G46710, ROPGAP3	Involved in cell wall patterning
Solyc09g009970.3	Rho GTPase-activating protein 1	0.80	2.33	AT3G11490, ROPGAP4	Involved in cell wall patterning

Solyc11g069590.2	Receptor-like serine/threonine-protein kinase NCRK	1.22	2.08	AT2G28250, NCRK	Necessary for basal callose deposition and ROS-dependent plasmodesmata (PD) closure
Solyc06g082610.4	Receptor-like kinase	0.85	1.97	AT5G05160, RUL1	Activate secondary growth, the production of secondary vascular tissues
Solyc01g111570.3	Proline-rich receptor-like protein kinase PERK7 isoform X1	0.88	4.80	AT3G05140, RBK2	ROP binding protein kinases 2
Solyc06g074070.3	Receptor-like kinase TMK3	0.62	5.61	AT2G01820, TMK3	Act as critical modulators of cell expansion and cell proliferation
Solyc05g054680.1	Receptor-like protein kinase THESEUS 1	0.91	2.58	AT5G54380, THE1	Encode THESEUS1 (THE1), a receptor kinase required for cell elongation during vegetative growth
Solyc09g030450.3	Receptor-like kinase	0.65	6.90	AT5G05160, RUL1	Activate secondary growth, the production of secondary vascular tissues
Solyc02g069970.4	Receptor-like protein kinase	1.21	4.05	AT1G30570, HERK2	Required for cell elongation during vegetative growth
**Others (promiscuous/poorly characterized substrates)**					
Solyc06g074670.4	UDP-apiose/UDP-xylose synthase	0.89	4.13	AT1G08200, AXS2	Synthesize sugar donors for cell wall polymers
Solyc11g066720.3	UDP-apiose/UDP-xylose synthase	0.82	3.93	AT1G08200, AXS2	Synthesize sugar donors for cell wall polymers
Solyc08g082250.3	Endo-beta-1,4-D-glucanase (*Cel8*)	1.07	3.10	AT1G64390, GH9C2	Cleave internal β-1,4-glycosidic bonds in cellulose and other β-glucans
Solyc09g010210.3	Endo-1,4-beta-glucanase precursor (*Cel2*)	0.95	0.06	AT1G70710, GH9B1	Cleave internal β-1,4-glycosidic bonds in cellulose and other β-glucans
Solyc04g081300.4	Endoglucanase	0.63	2.75	AT1G19940, GH9B5	Cleave internal β-1,4-glycosidic bonds in cellulose and other β-glucans
Solyc07g049300.3	Endoglucanase	0.84	18.55	AT2G32990, GH9B8	Cleave internal β-1,4-glycosidic bonds in cellulose and other β-glucans
Solyc09g075550.3	Cellulose synthase	0.57	3.89	AT1G02730, CSLD5	Mediate de novo cell wall formation
Solyc12g014430.2	Cellulose synthase	1.31	2.49	AT4G23990, CSLG3	Protein similar to cellulose synthase
Solyc01g058270.3	O-acetyltransferase family protein	0.99	2.20	AT3G06550, RWA2	Involved in polysaccharide O-acetylation in primary cell walls
Solyc02g071120.3	O-acetyltransferase family protein	0.91	2.49	AT2G34410, RWA3	Involved in cell wall acetylation
Solyc01g074030.3	Beta-glucosidase	1.08	2.54	AT2G44480, BGLU17	Hydrolyze β-glycosidic bonds to release glucose
Solyc01g081160.4	Beta-glucosidase 11	1.00	0.16	AT1G02850, BGLU11	Hydrolyze β-glycosidic bonds to release glucose
